# Molecular architecture of potassium chloride co-transporter KCC2

**DOI:** 10.1038/s41598-017-15739-1

**Published:** 2017-11-28

**Authors:** Morgane Agez, Patrick Schultz, Igor Medina, David J. Baker, Matthew P. Burnham, Ross A. Cardarelli, Leslie C. Conway, Kelly Garnier, Stefan Geschwindner, Anders Gunnarsson, Eileen J. McCall, Alexandre Frechard, Stéphane Audebert, Tarek Z. Deeb, Stephen J. Moss, Nicholas J. Brandon, Qi Wang, Niek Dekker, Anass Jawhari

**Affiliations:** 1 CALIXAR, 60 avenue Rockefeller, 69008 Lyon, France; 2 Department of Integrated Structural Biology, IGBMC (Institut de Génétique et de Biologie Moléculaire et Cellulaire) INSERM, U964; CNRS/Strasbourg University, UMR7104 1, rue Laurent Fries, BP10142, 67404 Illkirch, France; 3 INMED, INSERM Unité 901, Marseille, France; 4 Discovery Sciences, IMED Biotech Unit, AstraZeneca, Cambridge, UK; 5 Discovery Sciences, IMED Biotech Unit, AstraZeneca, Alderley Park, UK; 6 AstraZeneca Tufts Laboratory for Basic and Translational Neuroscience, Boston, Massachusetts, 02111 USA; 7 Discovery Sciences, IMED Biotech Unit, AstraZeneca, Gothenburg, Sweden; 8 Aix Marseille Univ, CNRS, INSERM, Institut Paoli-Calmettes, CRCM, Marseille Protéomique, Marseille, France; 9 Department of Neuroscience, Tufts University School of Medicine, Boston, MA 02111 USA; 10 Department of Neuroscience, Physiology and Pharmacology, University College, London, WC1E, 6BT UK; 11 Neuroscience, IMED Biotech Unit, AstraZeneca, Boston, MA USA

## Abstract

KCC2 is a neuron specific K^+^-Cl^−^ co-transporter that controls neuronal chloride homeostasis, and is critically involved in many neurological diseases including brain trauma, epilepsies, autism and schizophrenia. Despite significant accumulating data on the biology and electrophysiological properties of KCC2, structure-function relationships remain poorly understood. Here we used calixarene detergent to solubilize and purify wild-type non-aggregated and homogenous KCC2. Specific binding of inhibitor compound VU0463271 was demonstrated using surface plasmon resonance (SPR). Mass spectrometry revealed glycosylations and phosphorylations as expected from functional KCC2. We show by electron microscopy (EM) that KCC2 exists as monomers and dimers in solution. Monomers are organized into “head” and “core” domains connected by a flexible “linker”. Dimers are asymmetrical and display a bent “S-shape” architecture made of four distinct domains and a flexible dimerization interface. Chemical crosslinking in reducing conditions shows that disulfide bridges are involved in KCC2 dimerization. Moreover, we show that adding a tag to the C-terminus is detrimental to KCC2 function. We postulate that the conserved KCC2 C-ter may be at the interface of dimerization. Taken together, our findings highlight the flexible multi-domain structure of KCC2 with variable anchoring points at the dimerization interface and an important C-ter extremity providing the first in-depth functional architecture of KCC2.

## Introduction

Fast inhibitory neurotransmission in the central nervous system (CNS) is mediated by GABA and glycine via ligand-gated Cl^−^ channels. KCC2 is a neuron specific potassium-chloride co-transporter that is critically implicated in several neurological diseases including brain trauma, certain types of epilepsies and neuropathic pain^1^. KCC2 extrudes chloride, maintains low intracellular chloride concentration and renders GABA and glycine action hyperpolarizing in mature neurons^2–6^. The molecular mechanism of KCC2 function is not fully understood. Based on hydropathy analysis of the KCC2 protein, a model of KCC2, including 12 transmembrane domains flanked by intracellular N- and C-termini was proposed^7^. Although the information on the functional importance of the different structural elements of the KCC2 protein is limited, numerous studies suggest an important role of the C-terminus in control of KCC2 activity^8^. This co-transporter was shown to exhibit various posttranslational modifications (PTM) such as glycosylations and phosphorylations linked to function^1,9–15^. In terms of molecular assembly, KCC2 can form oligomers in an age-dependent manner as immature brains were characterized by a higher content of monomers, whereas adult tissue produced mostly oligomeric forms of KCC2^9^. An additional set of data demonstrating the ability of KCC2 to form dimers *in vivo* came from studies describing the distribution^16^ and interaction^17^ between KCC2a and KCC2b isoforms^18^. Uvarov and colleagues (2009) have shown that KCC2a and KCC2b isoforms form homo- and heterodimers *in vivo* in heterologous HEK293 cells. Despite the high interest in this protein due to its involvement in different neurological indications, structure-function of KCC2 remains poorly understood. To this end, it is critical to produce large amount of functional purified KCC2 to investigate its assembly, PTM and structure. Here, we characterized a set of tagged human KCC2 constructs and found that the addition of peptide tags to the C-terminus of KCC2 completely abolished its transporter activity. This first functional architecture of KCC2 will certainly serve for structure based drug design, fragment based drug discovery and antibody development towards the treatment of CNS disorders.

## Results

### Functional expression of KCC2

**Figure 1:**
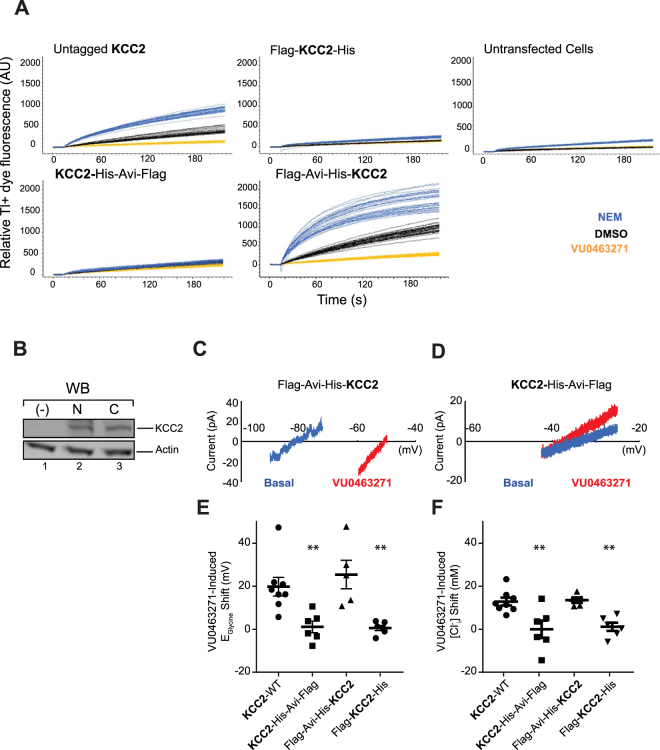
Thallium (Tl^+^) flux, expression of KCC2 constructs, and electrophysiology. (**A**) Thallium flux of untagged KCC2, N-terminal tagged KCC2 (Flag-Avi-His-KCC2), C-terminal tagged KCC2 (KCC2-His-Avi-Flag), double tagged KCC2 (Flag-KCC2-His) and untransfected cells are shown. Blue = NEM 100 µM, Black = DMSO 0.5%, Yellow = VU0463271 10 µM. Multiple blue lines are multiple NEM-treated wells (n = 32 for each treatment condition). (**B**) SDS-PAGE and Western-blot using antibody against KCC2 or actin as loading control (Sigma A5441 at 1:1000 dilution) in untransfected (lane 1), Flag-Avi-His-KCC2 transfected (lane 2) or KCC2-His-Avi-Flag transfected (lane 3) HEK293 cells. 20 µg per lane were loaded. (**C**) Example leak-subtracted traces from a Flag-Avi-His-KCC2 expressing HEK293 cells before (blue) and after (red) four-minute application of 10 µM VU0463271. (**D**) Example leak-subtracted traces before (blue) and after (red) VU0463271 application in a KCC2-His-Avi-Flag expressing HEK293 cell. (**E**) Summary data showing comparable shifts in E_GLY_ and (**F**) [Cl^−^] in KCC2-WT and Flag-Avi-His-KCC2, with no consistent shift observed in the KCC2-His-Avi-Flag-KCC2 or Flag-KCC2-His tagged construct expressing cells. **Indicates a significant difference (p < 0.01) in distribution from KCC2-WT. The chloride concentrations were calculated with the Nerst equation (see experimental procedure).

In order to produce a large amount of purified KCC2, we expressed different constructs of the wild type sequence (Untagged KCC2, Flag-KCC2-His, Flag-Avi-His-KCC2 and KCC2-His-Avi-Flag) in HEK293 cells. To evaluate the functionality of the wild type sequence constructs, we used the thallium (Tl^+^) flux assay. As previously described^19^, this method is based on the use of Tl^+^ as a potassium (K^+^) tracer. This assay relies on the ability of KCC2 to act either in influx or efflux mode according to the ion gradients present, combined with the ability of thallium to be transported by potassium transporters. Cells challenged with a high concentration of Tl^+^ and K^+^ were used to assess KCC2 influx activity using a fluorescent assay readout. Fluorescence is generated when Tl^+^ binds to a pre-loaded Tl^+^ sensitive dye inside the cell. HEK293 cells expressing untagged or amino-terminal Flag-Avi-His-tagged KCC2, displayed Tl^+^ flux in this assay (Fig. 1A). The flux could be reduced to background levels comparable to untransfected cell by the addition of the KCC2-specific transporter inhibitor VU0463271 (N-cyclopropyl-N-(4-methyl-2-thiazolyl)-2-[(6-phenyl-3-pyridazinyl) thioacetamide). N-ethylmaleimide (NEM) is known to stimulate KCC2 activity and addition of NEM to these cells resulted in an increase of Tl^+^ flux. The enhanced Tl^+^ flux was specific to KCC2, with no effect of NEM to untransfected cells (Fig. 1A). Interestingly, carboxy-terminal tagged KCC2 did not exhibit any basal activity and was not potentiated by NEM in the thallium assay. Thus, the addition of purification tags to the carboxy terminus completely abolished transporter activity. To rule out the possibility that these differences in activity are due to differences in expression, we analyzed the expression levels by Western-blot. Interestingly, transfected HEK293 cells for both N- and C-ter constructs showed similar KCC2 signal in Western-blot. No signal was observed for KCC2 in untransfected cells. Actin was used as loading control and shows the same signal in Western-blot in all conditions (Fig. 1B). To confirm these results, we used gramicidin perforated-patch clamp electrophysiology in transfected HEK293 cells. We assessed the functional expression of each construct by measuring VU0463271-induced shifts in the reversal potential of glycine-activated currents (E_GLY_) and the calculated [Cl^−^] values. Both the KCC2-WT and Flag-Avi-His-KCC2 exhibited VU0463271-induced positive shifts of E_GLY_ values (Fig. 1C) but no VU0463271-induced shifts were observed in cells expressing the KCC2-His-Avi-Flag (Fig. 1D) construct. Statistical analysis of E_GLY_ shifts showed a major effect of the construct (Fig. 1E; ANOVA: F = 9.23, *p* < 0.001) with KCC2-His-Avi-Flag (*p < *0.01) and Flag-KCC2-His (*p < *0.01), but not Flag-Avi-His-KCC2 (*p* = 0.66, ns), significantly different from KCC2-WT. These differences were reflected in analysis of [Cl^−^], with a major effect of the transfected construct (Fig. 1F; ANOVA: F = 8.50, *p* < 0.001) with KCC2-His-Avi-Flag (*p* < 0.01) and Flag-KCC2-His (*p* < 0.01), but not Flag-Avi-His-KCC2 (*p* = 0.99). Consistent with these observations, KCC2-WT and Flag-Avi-His-KCC2 expressing cells exhibited chloride extrusion upon washout of VU0463271, further indicating that they are functional (data not shown). KCC2 is known to reside both as a plasma membrane localized fraction and as a larger pool inside the cell in internal membranes^20^. To rule out the possibility that differences in KCC2 activity may correlate with differences in membrane localization, we performed immunofluorescence staining of different tagged version of KCC2. Figure S10 shows no alterations in KCC2 localization with the different constructs. This was also shown for Cos7 transfected cells (data not shown). These data indicate that the N-terminal tagged KCC2 construct is functionally active when expressed in HEK293 cells and was therefore selected for further biochemical and structural characterization.

### Solubilization and purification of KCC2

**Figure 2:**
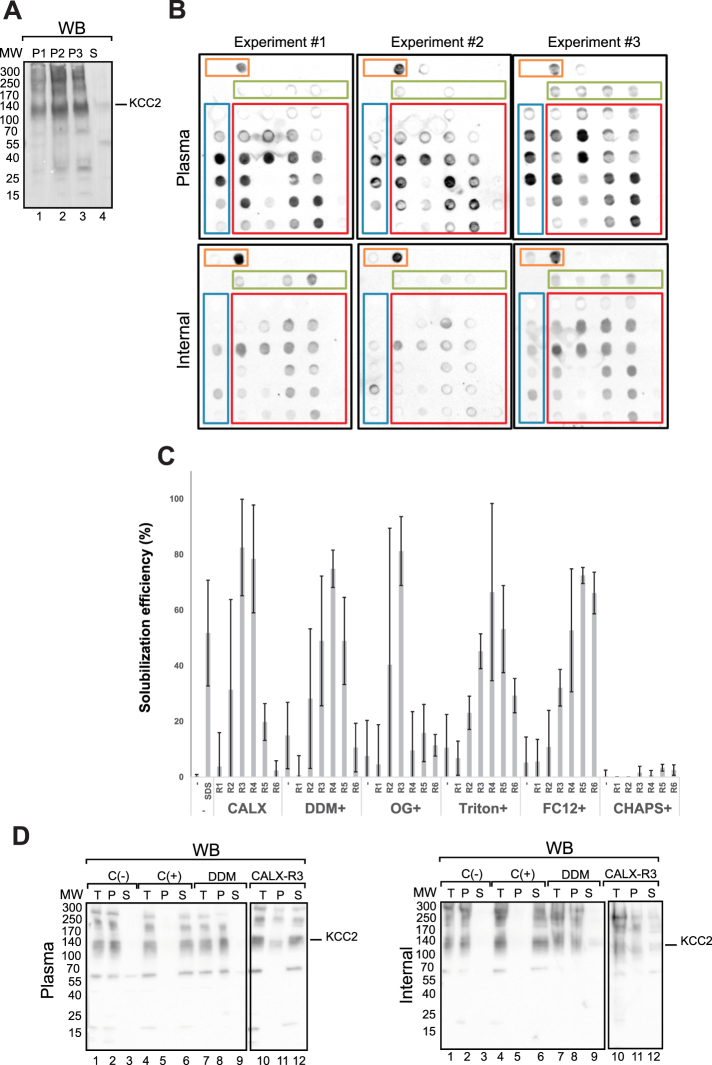
Fractionation and solubilization of native KCC2 from plasma and internal membranes. (**A**) Presence of KCC2 in cell membranes was detected by Western-blot using KCC2 specific antibody after membrane fractionation. Cell membrane were separated using sequential centrifugations after lysis of HEK293 cells. P1, pellet after centrifugation at 1000 × g (cell debris, whole cells); P2, pellet after centrifugation at 15000 × g (ER, mitochondria, nucleus); P3, pellet after centrifugation at 100000 × g (plasma membranes); and S, supernatant after centrifugation at 100000 × g. (**B**) Solubilization screen of KCC2 from internal and plasma membranes monitored by dot blot using KCC2 specific antibody. 10 critical micellar concentration (CMC) of each detergent, classical (in green, DDM, OG, Triton, FC12 and CHAPS) and calixarenes based detergents (in blue, CALX- R1-6), alone or in combination (in red) were used. Solubilization without or with SDS served as negative or positive controls, respectively (in orange). The experiment was done 3 times (**C**) Solubilization efficiency representation from relative intensity quantification of dot blot using Image Lab 4.1 software (Bio-Rad). Only dot blot from plasma membrane fractions were represented. All signal intensities are normalized to the highest signal observed for each dot blot (considered as 100%), n = 3. (**D**) Classical solubilization of internal and plasma membranes using selected conditions from dot blot screening followed by SDS-PAGE and Western-blot using a specific KCC2 antibody. Proteins from Total extract (T), pellets (P) and supernatants (S) after centrifugation at 100000 x g were analyzed. C(−) and C(+) correspond to the buffer (without detergent) and SDS, respectively.

**Figure 3:**
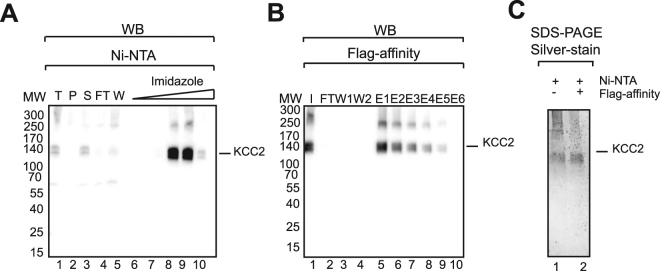
Purification of KCC2. CALX-R3 solubilized KCC2 (Flag-Avi-His-tagged on N-ter) was purified by (**A**) Ni-NTA chromatography followed by (**B**) Flag-affinity (Flag-M2 magnetic beads) and analyzed by SDS-PAGE and Western-blot (**A**,**B**) or silver stain (**C**). T: Total extract, P: Pellet after centrifugation at 100000 × g, S: Supernatant after centrifugation at 100000 × g, I: Input, FT: Flow-Through, W: Wash, E: Elution.

Flag-Avi-His-KCC2 (N-KCC2) was produced by transient transfection of HEK293 cells at large scale (20 Liters of culture). Cells were lysed by mechanical disruption. Membrane fractionation was performed using sequential centrifugation steps at 1000, 15000 and 100,000 x G corresponding to non-lysed cells, enriched internal membranes and enriched plasma membranes, respectively. The presence of KCC2 in each fraction was analyzed by SDS-PAGE followed by Western-blot using an antibody against KCC2 (Fig. 2A). Two main bands were observed at ~140 kDa and ~300 kDa in all fractions. These results indicate that KCC2 was present both in the internal and plasma membranes. The observed molecular weight of KCC2 of ~140 kDa, is higher than the theoretical molecular weight of ~120 kDa. These ~140 kDa band may correspond to different post-translationally modified populations of KCC2. This is consistent with previous reports showing that the glycosylated form of KCC2 migrates at ~140 kDa^9,10^. The band at 300 kDa may correspond to non-dissociated dimers that are SDS-resistant, which is often observed when membrane protein samples are not heated prior to gel migration to avoid aggregation and precipitation^21,22^. Since KCC2 was detected in internal and plasma membrane fractions, detergent screening using classical detergents (DDM, OG, Triton, FC12 and CHAPS), novel calixarenes based detergents (CALX reagents R1 to R6) or a combination of both was applied to both membrane fractions and results were assessed by dot blot using a KCC2 specific antibody. Figure 2B shows striking differences in KCC2 solubilization between internal and plasma enriched membrane fractions. Indeed, KCC2 from internal membranes was difficult to extract. This may correspond to a misfolded fraction due to KCC2 overexpression. On the contrary, plasma membrane KCC2 showed good solubilization mainly when calixarene based detergents were used alone (indicated in blue, Fig. 2B and C) or in combination with classical detergents (indicated in red in Fig. 2B and C). Plasma membrane solubilization efficiency is indicated by relative intensity quantification of dot blot (Fig. 2C). Dot blot analyses were used to generate first solubilization indications that needed to be further confirmed. Western-blot analysis confirmed the dot blot results (Fig. 2D). Human KCC2 was efficiently solubilized using CALX-R3 (~80% of solubilization yield, Fig. 2D, compare lanes 12 to 10) in contrary to DDM. Interestingly, similar solubilization conditions were obtained on mouse KCC2 expressed in the neuroblastoma cell line N2a or HEK293 cells (Figure S1). This demonstrates that KCC2 solubilization relies upon intrinsic common structural features of the 12 TM domains of KCC2 and not on the lipidic composition and/ or expression system diversity. We therefore focused on enriched plasma membranes and decided to use CALX-R3 as the solubilization reagent for subsequent purification steps. To avoid any possible detergent interference with the affinity column used for protein purification, we applied a minimal detergent concentration of 6 CMC (9 mM) which resulted in a KCC2 solubilization of ~80% (data not shown). We decided to use 9 mM of CALX-R3 as it was a good compromise between solubilization and detergent concentration. Solubilized material was loaded on His-tag affinity resins (Talon and Ni-NTA). The best results were obtained using Ni-NTA for which total binding and efficient elution of KCC2 were observed (Fig. 3A **)**. In contrast, talon resin did not result in good protein elution (Figure S2A). The purity obtained using Ni-NTA was relatively good (~70%) (Fig. 3C, lane 1) and Flag- affinity was applied as a second affinity step to improve purity. Figure 3B shows that the protein could bind to magnetic Flag-M2 beads and elute specifically from the resin. This was not the case for agarose Flag M2 resin (Figure S2B). Combining Ni-NTA and Flag-M2 purification, improved the purity to ~90% as shown in Fig. 3C (compare lane 2 to 1). In summary, KCC2 was solubilized using CALX-R3 from enriched plasma membranes fractions and isolated to a high purity using a tandem affinity purification (Ni-NTA and Flag).

### Characterization of the oligomeric state and the post-translational modifications of purified KCC2

**Figure 4:**
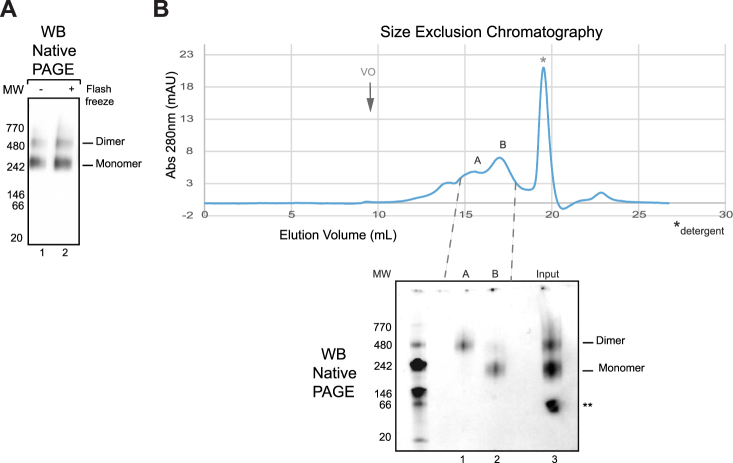
Homogeneity and stability of purified KCC2. Purified KCC2 was subjected (+) or not (−) to flash-freezing in liquid nitrogen and then (**A**) loaded on a 4–15% Tris-glycine Clear Native PAGE and detected by KCC2 antibody. (**B**) Gel filtration profile and corresponding Native PAGE revealed by KCC2 antibody show the separation of dimers and monomers of KCC2. *Indicates detergent migration in gel filtration. **Corresponds to nonspecific signal not observed in other Native PAGE gels. Void volume is indicated by an arrow and Vo.

**Figure 5:**
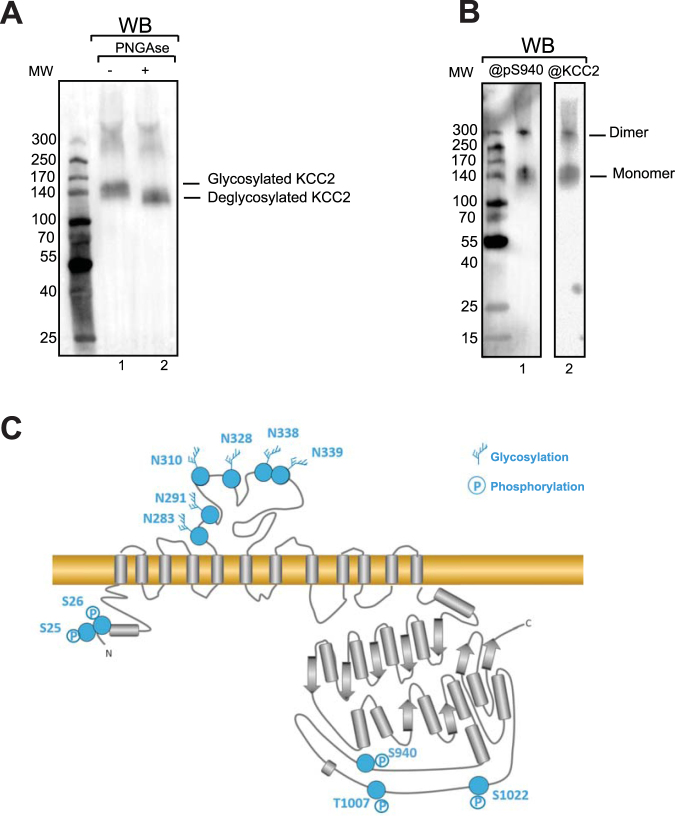
Post-translational modifications of KCC2. (**A**) SDS-PAGE and Western-blot of purified KCC2 treated or not with PNGase F using specific antibody against KCC2. (**B**) SDS-PAGE and Western-blot of purified KCC2 using an antibody directed against KCC2 Phosphoserine S940 (@pS940) or a specific anti-KCC2 antibody (@KCC2). Bands corresponding to KCC2 monomer and “SDS resistant” dimer are indicated. (**C)** Summary of mass spectrometry results on PTMs investigation. Secondary structure and topology predictions are indicated.

Several members of the cation-chloride cotransporter (CCC) family including KCC2 were reported to forms oligomers *in vivo* ^23,24^. To address whether KCC2 purified from HEK293 plasma membrane fractions forms aggregates, oligomers or monomers, the protein was assessed by Native PAGE similarly to previously reported work^25^. Two bands of KCC2 were observed on a Native PAGE (Fig. 4). They may correspond to the monomer and dimer, according to the molecular weight of protein markers. No aggregates were observed on Native PAGE even after a freeze-thaw cycle in the absence of a cryoprotectant. This illustrates the relative stability of KCC2 when solubilized and purified using the CALX-R3 compound. Interestingly, recombinant mouse KCC2 purified from HEK293 cells using the same solubilization and purification procedures, showed a virtually identical Native PAGE profile with a monomer and dimer band, but no aggregates and/ or higher oligomers (Figure S3) suggesting a common molecular assembly conserved between mouse and human KCC2. Gel filtration analysis confirmed a non-aggregated state of purified human KCC2 with no KCC2 in the void volume fraction and a separation between dimeric and monomeric populations shown by Native PAGE (Fig. 4B). The use of DDM to replace CALX-R3 reagent for gel filtration resulted in KCC2 aggregates (data not shown). Similar calixarene-based detergents were recently reported to stabilize native forms of membrane proteins^21,22,26–28^. Thus, we show that KCC2 purified using CALX-R3 compound exists as monomer and dimer and not higher oligomers or aggregates. To better characterize post-translational modifications of KCC2 and to evaluate its glycosylation state, purified protein was subjected to PNGase treatment. Figure 5A shows that KCC2 displayed increased mobility after PNGase digestion, demonstrating that purified KCC2 was glycosylated. To evaluate if KCC2 was phosphorylated at well-known residue serine 940, we used a specific antibody recognizing phospho-serine 940 (pS940)^13,14^. Figure 5B shows that purified KCC2 was recognized by this antibody similarly to KCC2 antibody. Both KCC2 and pS940-KCC2 antibodies reveal monomeric and dimeric KCC2 bands suggesting that both monomeric and dimeric KCC2 were phosphorylated at serine 940. In order to collect more information about KCC2 post-translational modifications, we analyzed purified KCC2 by mass spectrometry after trypsin digestion (Tables S4, Figures S5 and S6). A 44% KCC2 sequence coverage was obtained. Detected peptides are listed in Figure S5. It is possible that some of the non-detected peptides correspond to transmembrane helices that are difficult to detect by mass spectrometry. As suggested by Western-blot analysis, mass spectrometry reveals a heavily glycosylated KCC2 consistent with the observed gel shift after PNGase treatment (Fig. 5A). PNGase treatment and mass spectrometry analysis resulted in the identification of six glycosylation sites for N283, N291, N310, N328, N338 and N339 **(**Fig. 5C, Table S4, Figures S5 and S6). N291, N310 and N339 are well conserved between KCC2 and KCC4 and were only identified for KCC4 (Table S4). Identified phosphorylation sites were S25, S26, S940, T1007 and S1022. Among those, S1022 was identified in rat tissues^29^. S25 and S26 were observed in mouse^30^. S940 and T1007 has been described and directly correlated to KCC2 function^11–14^ and confirmed by Western-blot in the present work (Fig. 5B). These mass spectrometry results show that KCC2 was modified post-translationally on key functional residues.

### Functional characterization of purified native KCC2

**Figure 6:**
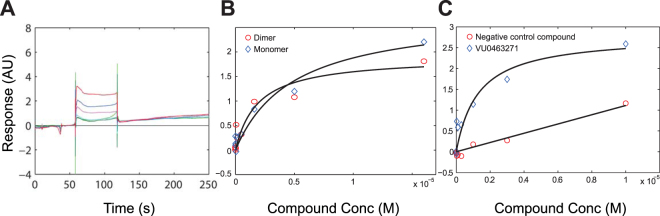
SPR ligand binding experiments with VU0463271 and KCC2 dimer and monomer. (**A**) Sensorgrams for binding of VU0463271 injected over purified KCC2 sensor surface in a 3-fold dilution series (see experimental procedure). (**B**) Concentration-dependant binding of VU0463271 on immobilized purified KCC2 dimer (red circles) and monomer (blue). Langmuir fit yield Kd (dimer) = 4 ± 2 µM (n = 2) and Kd (monomer) = 11 ± 7 µM (n = 2). (**C**) Concentration-dependent binding of VU0463271 (blue diamonds) and non-specific control compound (red circles) on immobilized purified KCC2. Langmuir fit yield Kd = 3 ± 2 µM (n = 3).

Given the critical role that KCC2 plays in neuronal function and the growing interest in generating novel selective blockers or activators, we decided to investigate binding of small molecule compounds to purified KCC2. We took advantage of the N-terminal His-tag of KCC2 for immobilization on a Ni-NTA chip to evaluate binding of a selective inhibitor VU0463271. This compound has been previously shown to selectively inhibit KCC2 leading to hyperexcitability and epileptiform discharges in the hippocampal slices^31–33^. Purified KCC2 could be effectively captured and covalently tethered to the biosensor allowing to generate a stable sensor surface for subsequent ligand binding experiments. Ligand-binding competence of the tethered material was assessed with the antagonist VU0463271 in a concentration-response experiment. Sensorgrams for binding of VU0463271 demonstrates a clear saturation, and an affinity of 3–11 µM (Fig. 6). We investigated binding of the antagonist compound VU0463271 to immobilized monomers or dimers separated by gel filtration (Fig. 4B). No significant difference in binding was observed for KCC2 monomer and dimer (Fig. 6B). The specificity of the binding was assessed using non-specific compound (Fig. 6C). Thus, KCC2 shows indirect and direct functional evidence by Biacore and electrophysiology/ thallium assays, respectively.

### Structural organization of KCC2

**Figure 7:**
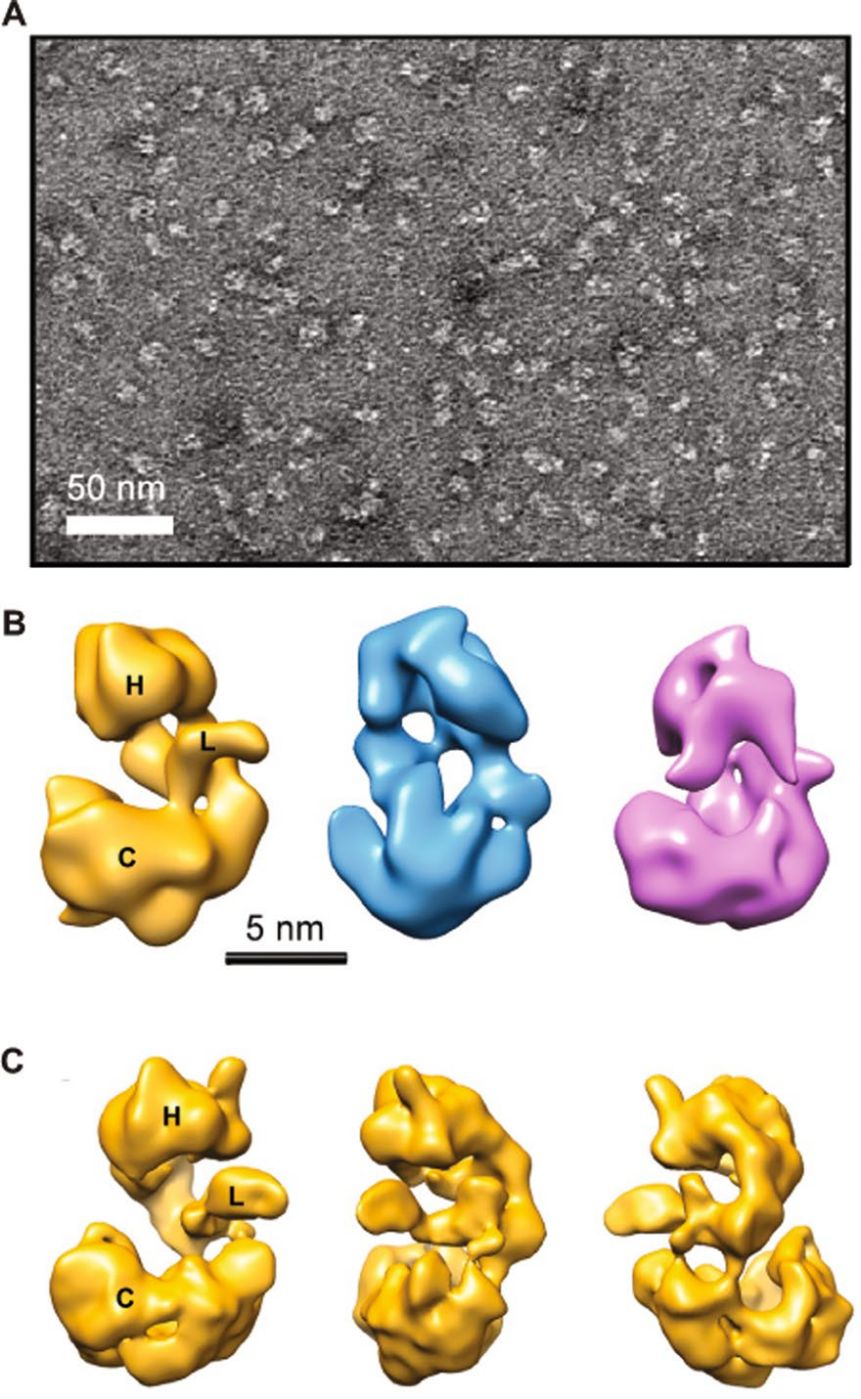
Electron microscopy of KCC2 monomers. (**A**) Negative stain image showing particles of purified KCC2 monomers of about 90–120 Å in size and (**B**) 3D class averages of monomers. Classes I, II and III correspond to 30, 32 and 38% of the monomeric particles, respectively. (**C**) 3D class I in which the two domains were best resolved is presented in different views after refinement. Head, core and linker structures are indicated by H, C and L, respectively.

**Figure 8:**
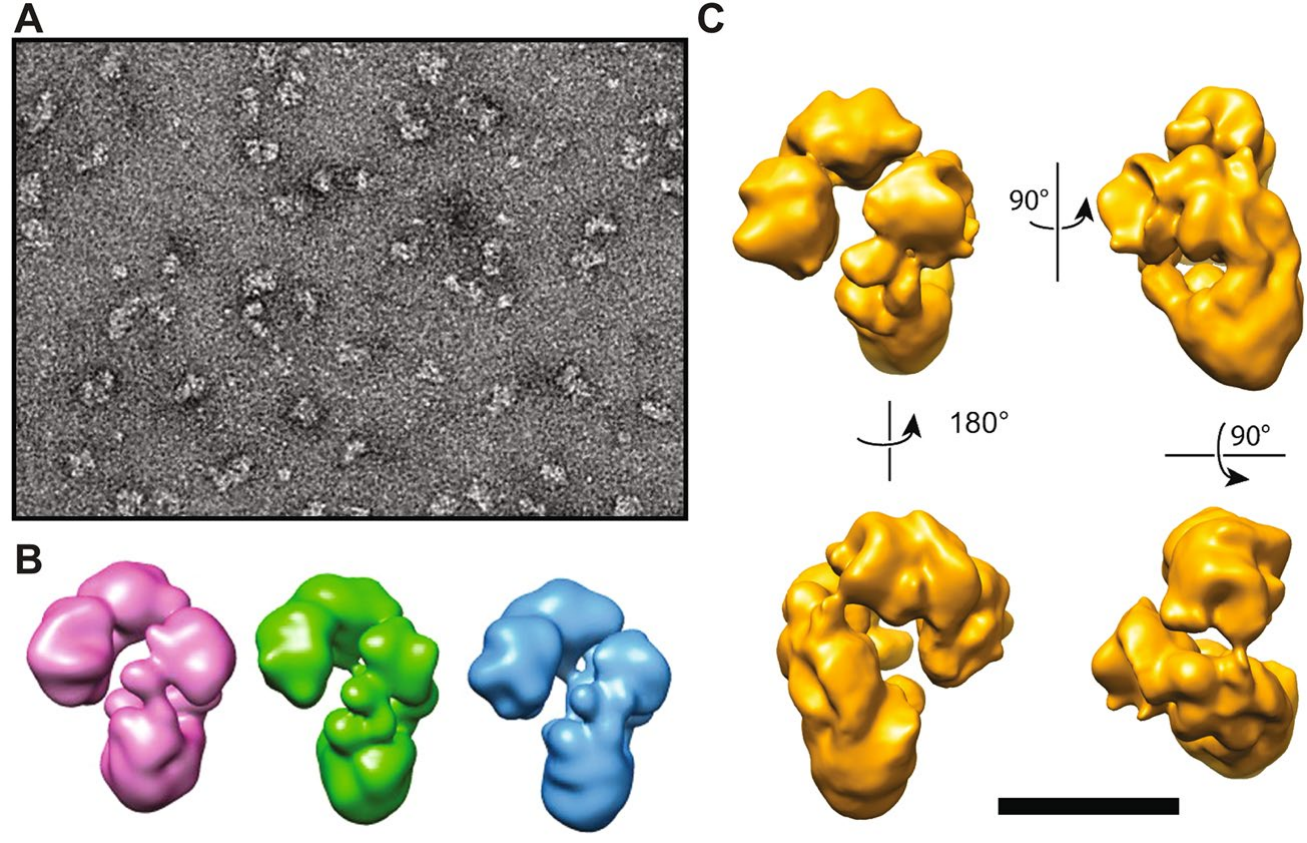
Electron microscopy of KCC2 dimers. (**A**) Negative stain image showing particles of purified KCC2 dimers of about 150–180 Å in size and (**B**) 3D class averages of dimers. Classes I, II and III correspond to 30, 32 and 38% of the dimeric particles, respectively. (**C**) Different views of refined 3D model are shown. The scale bar corresponds to 100, 12.5 and 11 for (**A**,**B** and **C**), respectively.

**Figure 9:**
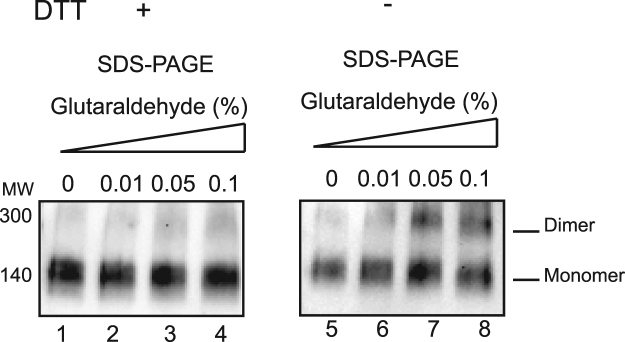
KCC2 chemical crosslinking. Increasing amount of glutaraldehyde was added to purified KCC2 in presence or absence of DDT and analyzed by SDS-PAGE and Western-blot.

In order to investigate the structural organization of purified KCC2, we have used negative stain electron microscopy (EM) to analyze the gel filtration fractions of KCC2 monomers and dimers. When observed by EM, the KCC2 monomer fraction shows isolated particles about 90–120 Å in size (Fig. 7A **)**. A total of 2000 micrographs were recorded and 114,370 particles were selected, aligned and clustered into classes. The resulting class averages show different views of the KCC2 monomer which often appears to be formed by two domains. (Figure S7-A). A 3-D model was calculated ab initio and the image dataset was clustered into 3 classes to reveal heterogeneities or conformational changes of the sample **(**Fig. 7B). The three classes are slightly elongated and are organized into two major domains connected by a linker domain. The smallest “head” domain appears to be flexible and adopts different positions as compared to the largest “core” domain. Classes I, II and III correspond to 30, 32 and 38% of the monomeric particles, respectively, suggesting that the conformational changes are continuous. Class I in which the two domains were best resolved was further refined to reach a resolution of ~15 Å **(**Fig. 7C and video S9) as evaluated by Fourier Shell Correlation curves. The EM observation of the KCC2 dimer fraction revealed particles about 150–180 Å in size, significantly larger than the monomers (Fig. 8A **)**. To gain more insights into the quaternary structure of dimeric KCC2, we recorded 2,000 micrographs and selected 78,781 dimeric particles, which were aligned and clustered into different classes. Figure S7-B shows the resulting class averages. A 3-D model was calculated ab initio from the dimeric particles and the image dataset was clustered into 3 classes to identify distinct conformations (Fig. 8C). As the three classes did not reveal significant conformational changes, the whole dataset was refined to reach a 3-D model with a resolution of ~17.4 Å (Fig. 8B and video S8). The 3-D model of the dimeric fraction has the same overall architecture, consisting of 4 different structural modules connecting to each other to form an elongated bent “S” shape. A flexible “hinge” region observed in all classes may account for a very flexible anchoring dimerization interface between monomers. To test the contribution of disulfide bridges to KCC2 dimerization, we incubated purified KCC2 with DTT and then subjected the sample to chemical crosslinking using glutaraldehyde. A good correlation between crosslinker concentration and the appearance of the KCC2 dimer band was observed (Fig. 9). Interestingly, the presence of DTT results in a significant reduction of dimerization suggesting that disulfide bridges are involved in KCC2 dimerization.

## Discussion

We show that KCC2 exists as monomers as well as dimers in solution but not trimers, tetramers or higher homo-oligomers as previously reported^9^. One explanation of this discrepancy may be due to misleading SDS resistant effect in SDS-PAGE. Our study did not base oligomeric state assessment on SDS-PAGE patterns but rather on Native PAGE where SDS-resistant complex artefacts are not observed. In addition, crosslinking experiments in reducing and non-reducing conditions, gel filtration and electron microscopy studies show the presence of monomers and dimers. Transporters such as NKCC1 demonstrated a dimeric organization^24,34,35^, which is consistent with our finding. Our Biacore experiments suggest that both the monomer and dimer are functional but further experiments such as mutagenesis of residues at the dimer interface will need to be performed to address this question.

**Figure 10:**
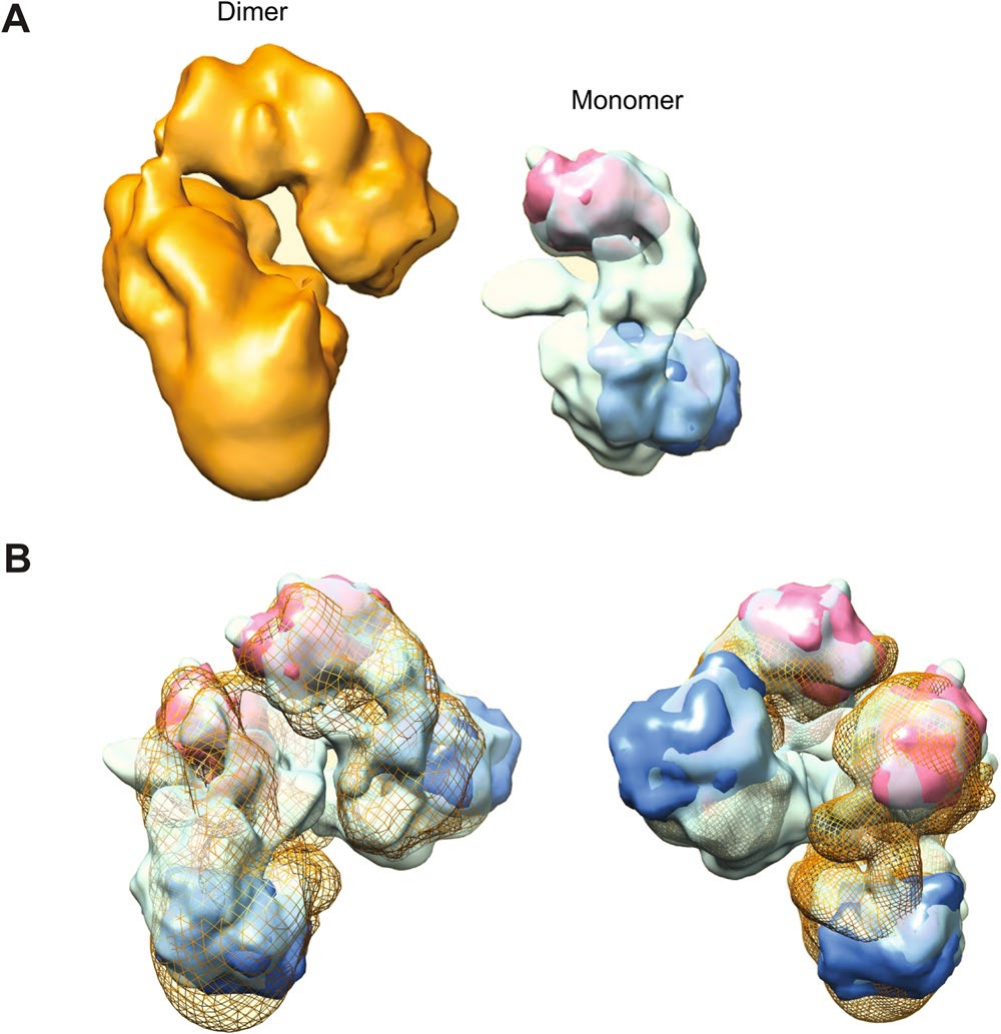
Architecture of KCC2. (**A**) KCC2 dimer and monomer are represented in orange and green, respectively. Glutamate GABA anti-porter structure (PDB: 4DJK) and prokaryotic cation-chloride cotransporter (PDB: 3G4O) were filtered to 12 Å and fitted manually followed by optimization of the fit using UCSF Chimera. (**B**) Proposed fit of two monomers into a dimer. Two views are represented (blue and red correspond to Glutamate GABA anti-porter and prokaryotic cation-chloride cotransporter, respectively). In mesh colored in orange is represented the surface of KCC2 dimer reconstruction.

Sequence analysis and topology predictions suggest that KCC2 possesses two different structural domains. The first could be composed of 12 transmembrane helices (TM) and the second corresponding to a carboxy-terminal cytoplasmic domain (C-ter). The first domain is homologous to the glutamate-GABA anti-porter structure (PDB: 4DJK), while the C-ter domain displays sequence/ secondary structure homology to the prokaryotic cation-chloride cotransporter (PDB: 3G4O). Figure 10A shows a fit of both structures filtered to 12 Å. Additional density remains unfitted and may account for linkers and long loops. Two monomers were found to fit well to the dimer (Fig. 10B). Other fits are certainly possible and higher resolution Cryo-EM structures will be required to better map KCC2 domain organization. Interestingly, the prokaryotic cation-chloride cotransporter was shown to dimerize in solution^36,37^ suggesting that the KCC2 C-ter domain may be at the interface of dimerization. Our study demonstrates that in contrast to KCC2 WT and the tagged in N-terminus KCC2, modification of KCC2 at the C-terminus by addition of a His-Avi-Flag-tag led to inactivation of the transporter in both thallium assays and in electrophysiology assays. Therefore, we propose that the dimerization interface is composed of at least two anchor points, one being mediated by disulfide bridges (perhaps between specific extracellular loops present on monomers) and the second maybe involving functional homodimerization of the C-ter domain. Figure 10B. Further studies on functional investigation (ligand binding and transport) of KCC2 C-terminal mutants are needed to confirm that. Purified KCC2 exhibits specific key posttranslational modifications such as glycosylation and phosphorylation that have been reported to be required for KCC2 cotransporter activity^13,14,25,31^. Here, the phospho-serine 940 at the intracellular C-terminal domain was specifically recognized by an antibody directed against the phosphorylated state of this residue. This was unambiguously confirmed by mass spectrometry. Previously reported phosphorylated residues such as S940 and T1007 could be confirmed in the present study. S1022 phosphorylation was previously reported for KCC4 and not for KCC2. Our study could identify specific glycosylation sites of KCC2. The exact functional role of each of these modifications will need to be further investigated.

This study describes the purification of homogenous, non-aggregated and functional native KCC2 using a calixarene-based detergent. These solubilizing and stabilizing compounds could facilitate the production of other challenging native membrane proteins such as GPCRs, transporters, ion channels or immunity proteins while maintaining their structural stability, homogeneity and functional integrities^21,22,26,28^. This study serves as a basis for the isolation of other membrane proteins of high importance to human health and for the discovery of novel compounds that could regulate the K^+^ Cl^−^ co-transporter activity of KCC2. This work will also serve as a starting point for cryo-electron microscopy and functional investigations to better understand KCC2 functional domains and to help delineate common mechanistic features within the cation chloride cotransporters family. As such, this work could inform novel therapeutic routes for epilepsy, pain and schizophrenia treatment.

## Experimental Procedures

### KCC2 Construct generation

The following constructs were expressed from an in house vector featuring a CMV promoter derived from pcDNA 3.1: 1) wild type KCC2 corresponding to residues 1–1116 of NP_065759 (human KCC2 isoform 2), 2) double tagged construct Flag-KCC2-His with additional DNA coding sequences for MDYKDDDDK added to the amino terminus and HHHHHHHHHH to the C-terminus (in pTT5), 3) N-terminal tagged Flag-Avi-His-KCC2 with coding sequence MGDYKDDDDKGGGLNDIFEAQKIEWHEGGHHHHHHHHHHGGG added to the N-terminus, and 4) C-terminal tagged KCC2-His-Avi-Flag with coding sequence GGGHHHHHHHHHHGGGLNDIFEAQKIEWHEGGDYKDDDDKG added after residue 1116.

### Transient transfection and cell culture

The various human KCC2 constructs were transfected into HEK293 cells using the MaxCyte STX electroporation. Expression of human KCC2 was confirmed by Western-blot using Rabbit anti-KCC2 as primary antibody (Sigma/Millipore #07-432). Culture media was DMEM (Sigma, D6546) with 10% FBS and 1% GlutaMAX (Gibco, 35050-038). For large scale production of protein for binding studies, suspension Expi293F cells were cultured in Expi293F media and transfected at high density with KCC2 plasmid DNA diluted to 1 mg/ml using PEIMax at 1 mg/ml^38^. Transfection was carried out on 250 ml of suspension cells at 4 × 10^6^/ml in vented 2 L capacity roller bottles shaken in an atmosphere of 5% CO_2_ at 37 °C and 140 rpm in an orbital incubator (25 mm orbit). DNA and PEIMax were pre-incubated in E293F media for 15 minutes before adding to the cells. Twenty-four hours post transfection, cultures were fed with 50% media making 500 ml total volume per roller bottle and incubated for a further 48hrs. Cell pellets were harvested 72hrs post transfection by centrifugation at 10,000 × g for 20 min and stored at −80 °C prior to purification. Cells and media were purchased from ThermoScientific; 40 kDa linear PEI Max as hydrochloride salt (catalogue #24765) from Polysciences. Mouse KCC2 was expressed in HEK293 and N2a cells as previously described^5,39–41^.

### Thallium assay

Following 48 hours incubation at 37 °C (5% CO_2_) cells were washed with HBSS (Hank’s Balanced Salt Solution). using a BioTek EL406 washer. Thallium assay buffer was made by diluting the Fluxor kit assay buffer (Life Tech #F10017) to 1X. Assay buffer was adjusted to pH 7.4 using NaOH. Loading buffer, which contained a fluorescent thallium sensitive dye (1000X), was formulated in a solution of Back Drop Suppressor (Life Tech #B10512) containing 10 µM bumetanide (Sigma #B3023) and 10 µM ouabain (Sigma #O3125) Cells were loaded with loading buffer using a Multidrop Combi After incubation at room temperature for 60 minutes, compounds were added using the FLIPR. Treatments consisted of DMSO (final conc. 0.5%), VU0463271 (^32^, 10 µM final conc.) and N-ethylmaleimide (NEM, 100 µM final conc.) to see maximum thallium flux. Following a 90 minutes incubation at room temperature, thallium stimulus buffer was added to the cells on the FLIPR and thallium flux recorded for 220 seconds (ex. 515–575 nm). Data was analyzed using Screenworks v3.2 software (Molecular Devices). Maximum signal minus minimum signal between 1–120 seconds was used to compare groups.

### Electrophysiology

Sensitivity to the specific KCC2 inhibitor VU0463271^31,32^ was assessed in HEK293 cells transiently transfected (Lipofectamine 2000 reagent) with the cDNA for GFP, GlyR1, and either WT KCC2b, His-Avi-Flag-KCC2, KCC2-His-Avi-Flag, or Flag-KCC2-His. Cells were incubated for 48 h prior to recording. Cell culture medium was DMEM containing 10% FBS and 1% Pen/Strep and cells were grown in a 5% CO_2_ atmosphere incubator at 37 °C. Recordings were conducted at room temperature (25 °C) in bath saline containing (in mM) NaCl 140, KCl 2.5, MgCl_2_ 2.5, CaCl_2_ 2.5, HEPES 10, glucose 11, pH 7.4 NaOH. Isolated GFP + HEK293 cells were selected by epifluorescence and perforated with gramicidin (50 mg/ml) in a patch pipette containing (in mM) KCl 140, HEPES 10, pH 7.4 KOH. All chemicals were purchased from Sigma Aldrich (Pittsburgh, PA) and all cell culture reagents were purchased from Invitrogen (Carlsbad, CA). Solutions were applied with a three-barrel microperfusion system (700 µm, Warner Instruments, Hamden, CT) positioned closely above the cell. After establishing a series resistance of < 100 MΩ, bumetanide (10 µM) was added and a stable initial equilibrium potential for glycine currents (E_GLY_) was found by application of glycine (50 µM) during positive-going voltage ramps (20 mV/1 s). The chloride concentrations were calculated with the Nerst equation, which relates observed equilibrium potential to concentrations of chloride on each side of the membrane. Because the outside concentration is controlled in the experiment, it can be solved for the internal concentration before and after application of VU0463271. Typically, E_GLY_ was calculated using linear-regression fits to leak-subtracted currents. Next, VU0463271 (10 µM) was added with glycine pulses (50 µM, 3 s duration) applied every 20 s for a total of four minutes, after which a voltage ramp was used to calculate shifts in E_GLY_. Data were acquired at 10 kHz with an Axopatch 200B amplifier and Clampex 10 software (Molecular Devices, Sunnyvale, CA). Differences between treatments were assessed by one-way ANOVA with Dunnett’s multiple comparison tests comparing each shift to that observed in KCC2-WT expressing cells. Data are expressed as mean ± SEM.

### Immunofluorescence Staining

HEK293 or Cos7 cells were plated on poly-L-lysine coated coverslips. Tagged KCC2 constructs were transfected into HEK293 or Cos7 cells using Lipofectamine 2000 reagent. After 48 hours, cells were fixed in 4% paraformaldehyde. Cells were then washed in PBS and blocked for 1 hour at room temperature (5% BSA, 5% Normal Goat Serum, 0.1% Triton X-100 in PBS). Following blocking, coverslips were incubated with a KCC2 primary antibody (Millipore 07–432, diluted 1:200 in 2.5% BSA, 5% Normal Goat Serum, 0.1% Triton X-100 in PBS) for 2 hours at room temperature. Coverslips were then washed in PBS and incubated for 1 hour at room temperature with a goat anti-rabbit secondary antibody conjugated to Alexa Fluor 568 (Invitrogen A-11036, diluted 1:500 in 2.5% BSA, 5% Normal Goat Serum, 0.1% Triton X-100 in PBS). Coverslips were then washed in PBS and mounted in ProLong Gold Antifade Reagent with DAPI (Life Technologies, P36931). Cells were imaged with a confocal microscope using a 60x objective. Identical laser settings were used when imaging constructs expressed in each cell type.

### Membranes fractionation

Cells expressing mouse or human KCC2 were thawed on ice for 1 h with PBS (25 mM Na2HPO4, 150 mM NaCl, Protease Inhibitor Cocktail 1X. Mechanical cell lysis was performed on ice using a BeadBeater homogenizer with 0.1 mm diameter glass beads, including 5 pulses of 30 s with 2 minutes break in between. Membrane fractionation was carried out at 4 °C by sequential centrifugations: 1000 × g for 5 min, 15000 × g for 30 min, and 100000 × g for 45 min. Internal (15000 × g fraction) and plasma membranes (100000 × g fraction) were resuspended in PBS, PIC 1X, 20% glycerol to a final concentration of 10 mg/ml, flash-frozen and stored at −80 °C.

### Protein solubilization

Proteins from internal or plasma membrane fractions were incubated for 2 h at 4 °C at a final concentration of 5 mg/ml BCA in 1X PBS and 1X protease inhibitor cocktail, using different detergents conditions (classical and calixarenes based detergents CALX-R1 to R6, alone or in combination) at 10 CMC (Critical Micelle Concentration) for all compounds to be able to compare their solubilization power. Extraction without detergent and with SDS served as negative and positive controls, respectively. Dot blot analysis was performed to evaluate the solubilization efficiency as described^21^. To confirm the results, solubilization was done again in similar condition using selected detergent, as well as increasing concentration of detergent. After solubilization samples were centrifuged at 100000 x g for 45 min at 4 °C and an aliquot of the total extract, the pellet and the supernatant from each solubilization condition was analyzed by SDS-PAGE and Western-blot. Membranes prepared from N2a or HEK cells expressing mouse KCC2, were solubilized as described here.

### Protein purification

#### His-tag affinity chromatography

The soluble protein fraction was loaded onto a TALON or Ni-NTA resin (40 μl of dry resin per mg of plasma membrane proteins). After 2 h incubation at 4 °C, resin was washed with 10 Column Volumes (CV) of Wash Buffer 1 (PBS 1x; CALX-R3, 9 mM), Imidazole 10 mM). Target protein was eluted with 4 times 3 CV of PBS 1x, DDM (0.6 mM), CALX-R3 (1.5 mM), with increasing concentration of Imidazole (20 mM, 50 mM, 100 mM, 250 mM). Samples of each fraction were analyzed by SDS-PAGE and Western-blot.

#### Flag-affinity chromatography

A tandem affinity chromatography was applied (His-tag followed by Flag-tag). Elution fractions from His-affinity chromatography were loaded on anti-FLAG affinity resin (40 μl of either 50% suspension of Anti-Flag M2 magnetic or agarose beads per mg of plasma membrane proteins) and incubated overnight at 4 °C. Resin was then washed successively with 3 times 10 CV of PBS 1x, CALX-R3 (1.5 mM), DDM (0.6 mM), FLAG peptide 0.2 mg/ml. Samples of each fraction were analyzed by SDS-PAGE and Western-blot. When needed, the eluted protein was concentrated using Amicon ultra 100KDa flash-frozen in liquid nitrogen and stored at −80 °C until use.

#### Size exclusion chromatography

50 μl of purified KCC2 at 1 mg/ml was loaded on a superdex 200 Increase 5/150 GL(GE-Healthcare) at 0.1 ml/min. Running buffer was PBS 1x, CALX-R3 (1.5 mM), DDM (0.6 mM). Elution was performed at 0.1 ml/min with 1.5 CV of running buffer and 150 µl-fractions were collected. Fractions were analyzed by CN-PAGE and Western-blot.

### SDS-PAGE and Western-blot

Purified hKCC2 was denatured with 5x Laemmli buffer and incubated for 5 min at RT prior to analysis without heating to avoid aggregates formation. Proteins were separated by SDS-PAGE on a 4–15% acrylamide gel (4–15% Mini-PROTEAN® TGX Stain-Free™ Gel, *Bio-Rad*) and subsequently immobilized by electro-transfer to PVDF membrane. The immunodetection of KCC2 was performed by using the SNAP i.d. system (*Millipore*) with either a primary goat anti-KCC2, a primary rabbit anti-phosphorylated S940, an anti-Flag horseradish peroxidase (HRP) conjugated or an anti-His HRP antibody, as specified in the text. Quantification of the signal was performed using Image Lab 4.1 software from *Bio-Rad* to calculate extraction efficiency.

### Silver Stain/Coomassie Stain

SDS-PAGE were silver stained using Bio-Rad Dodeca Silver Stain Kit following supplier protocol or coomassie stained using the PageBlue™ protein staining solution.

### Clear Native-PAGE (CN-PAGE) and Western-blot

Non-denaturated proteins were separated by Native-PAGE on a 4–15% acrylamide gel (4–15% Mini-PROTEAN^®^ TGX Stain-Free™ Gel, *Bio-Rad*) using 25 mM imidazole as anode buffer and 7.5 mM imidazole, 0.05% deoxycholate, 0.01% DDM as cathode buffer). Clear Native PAGE gels ran for 90 min at 200 V and 4 °C. Proteins were then immobilized by electro-transfer to PVDF membrane. The immunodetection of KCC2 was performed by using the SNAP i.d. system (*Millipore*) with either a primary goat anti-KCC2, an anti-Flag horseradish peroxidase (HRP) conjugated or an anti-His HRP antibody. For cryo-stability, aliquots of purified protein were submitted to freeze/thaw cycle in liquid nitrogen in absence of cryoprotectant then analyzed by CN-PAGE.

### Protein quantification

Total protein concentrations in the plasma and the internal membrane fractions were determined with the micro BCA protein assay kit (*Pierce*) using the bovine serum albumin (BSA) as a standard.

### Chemical crosslinking

Purified protein (5 µg) was submitted to crosslinking by addition of different concentration of glutaraldehyde 0, 0.01%, 0.02%,0.05%, 0.1% for 30 minutes at 4 °C. Samples were analyzed by SDS-PAGE and Western-blot.

### Post-translational modifications of KCC2

Presence of the phosphorylation on the Serine S940 of purified KCC2 protein was detected by Western-blot using a primary specific antibody against KCC2 Phospho-serine 940 (Interchim, #PLX360). Concerning glycosylation, purified KCC2 was subjected to deglycosylation using PNGase F (Glycopeptidase F) then analyzed by SDS-PAGE and Western-blot. SDS-PAGE bands containing glycosylated and deglycosylated KCC2 were cut and analyzed by mass-spectrometry. Mass spectrometry analyses were performed as already described^42^. Raw files generated from mass spectrometry analysis were processed with Proteome Discoverer 1.4 (ThermoFisher Scientific). This software was used to search data via in-house Mascot server (version 2.4.1; Matrix Science Inc., London, UK) against the variant 27A- >V; 77R- >Q of KCC2 isoform 2 added to the human subset (20, 160 sequences) of the SwissProt database (2016_10). Database searches were done using the following settings: a maximum of two trypsin miscleavages allowed; methionine oxidation, Serine/Threonine phosphorylation as variable modifications. A peptide mass tolerance of 6 ppm and a fragment mass tolerance of 0.8 Da were used for search analysis. Only peptides with high stringency Mascot score threshold (identity, FDR < 1%) were selected and used for protein identification. PhosphoRS 3.0 tool was activated in ProteomeDiscoverer software to enable automated and confident localization of phosphorylation sites within validated peptides sequences.

### Negative staining electron microscopy

Five microliters of purified KCC2 at 40 µg/ml (in gel filtration buffer, PBS 1x, CALX-R3 (1.5 mM), DDM (0.6 mM)) were adsorbed for 30 sec. on 200 Mesh copper grids coated with formvar-C and treated by a glow discharge in air for 2 min. The grids were stained with 2% uranyl acetate for 1 min. A total of 2000 image frames were recorded for both the nomomer and the dimer fractions on a Transmission Electron Microscope (Tecnai F20 G2, FEI) equipped with a field emission gun operating at 200 kV at a magnification of 66.000 x, on a 2048 × 2048 CCD camera (Ultrascan 1000, Gatan Inc., Pleasanton) with a final pixel spacing on the specimen of 0.212 nm. Particles were selected manually using the Boxer application in the EMAN software package^43^. Two-dimensional class averages were calculated using the Relion software package^44^ and further used to extract automatically the particle images. After several rounds of image sorting 78,781 dimer and 114,370 monomer particles were selected, aligned, clustered into different classes and class averages were calculated. The SIMPLE software^45^ was used to generate a starting 3-D model which was further refined with Relion using standard procedures. Figures were prepared using UCSF Chimera^46^.

### Surface Plasmon Resonance (SPR) binding experiments

The experiments were performed on a Biacore S200 optical biosensor unit (GE Healthcare) at 10 °C using sensor chips Series S NTA (Research grade). The running buffer was composed of 10 mM HEPES, 150 mM NaCl, 0.1% (w/v) DDM, 0.0024%(w/v) CHS, 0.012%(w/v) CHAPS, pH 7.4 and was delivered at a flow-rate of 20 µl/min. Prior to covalent tethering of KCC2 protein, the surface was conditioned by 1 min injection 10 µM NiCl2. The surface was activated for 7 min with 50 mM NHS and 200 mM EDC and the flowrate was subsequently reduced to 1 µl/min. KCC2 protein at a final concentration of 300 µg/mL was injected over the surface with a contact time of 20 min resulting in a final density of approximately 4000 RU. The reference surface was prepared identically with the exception of no protein added. The ligand binding experiments were performed at a flow rate of 30 µl/min using multi-cycle kinetics. The antagonist VU0463271 and negative control compounds was injected in concentration response experiments using a 3-fold dilution series at 0.5% DMSO. The equilibrium dissociation constant was determined by non-linear regression analysis of the steady-state SPR signals as a function of ligand concentration using the Langmuir isotherm equation.

## Electronic supplementary material

Supplementary Information

Video S8

Video S9
