# Supplementary material for: Molecular architecture of potassium chloride co-transporter KCC2
Source: Sci Rep. 2017 Nov 28;7:16452. doi: 10.1038/s41598-017-15739-1 (PMC5705597; doi:10.1038/s41598-017-15739-1)

TITLE: Molecular architecture of potassium chloride co-transporter KCC2.

AUTHORS:

Morgane AGEZ[1*], Patrick SCHULTZ[2*], Igor MEDINA[7*], David J. BAKER[4] , Matthew P BURNHAM[4], Ross CARDARELLI[5], Leslie C. CONWAY[5], Kelly GARNIER[1], Stefan GESCHWINDNER[3], Anders GUNNARSSON[3], Eileen J McCALL[4], Alexandre FRECHART[2], Stéphane AUDEBERT[8], Tarek DEEB[5], Steve MOSS[5], Nick BRANDON[6], Qi WANG[6], Niek DEKKER[3¶] & Anass JAWHARI[1¶]

SUPPLEMENTARY INFORMATION

Figure S1: Solubilization of mouse KCC2 (His-tagged) from plasma membranes of **A**- N2a cells and **B**- HEK cells**.** No detergent and SDS serve as negative (-) and positive (+) controls, respectively. Proteins from Total extract (T), pellets (P) and supernatants (S) after centrifugation at 100000g were analyzed by SDS-PAGE and western-blot using an anti-His tag antibody.

Figure S2: Affinity purification of human KCC2 using **A**- His-tag with Talon affinity versus Ni-NTA and B- Flag-tag with Flag M2 agarose resin versus Flag M2 magnetic beads. The results were monitored by SDS-PAGE and western blot using KCC2 antibody.

Figure S3: Solubilization, His-tag Talon affinity purification of mKCC2 expressed in HEK293 cells (**A**) and its oligomeric state (**B**)**.** Proteins from each solubilization and purification were separated by SDS-PAGE and analyzed by western-blot using a specific anti-KCC2 antibody. Purified KCC2 protein was loaded on CN-PAGE and detected by western blot using a specific anti-KCC2 antibody.

Table S4: List of experimental post-translational modifications found on KCC2 purified sample in comparison to previously reported work.

Figure S5: Sequence coverage of peptides (44 % of full length) of S12A5_HUMAN protein identified by LC-MSMS.

Figure S6: PTM Mass spectrometry data of KCC2.

Figure S7: Class averages of KCC2 **A**- monomers and **B**- dimers.

Video S8: Video of KCC2 dimer structure.

Video S9: Video of KCC2 monomer structure.

Figure S10: Immunofluorescence staining of tagged KCC2 constructs expressed in HEK293 cells. KCC2 staining is shown in red (left), DAPI staining is shown in blue (middle), and a merge of the two channels is shown (right). Images were acquired using confocal microscopy. Scale bars, 20µm.

Figure S11: Original gels or Western-blot used for **A**- Figure 1B, **B**- Figure 2D, **C**- Figure 5B and **D**- Figure 9. A rectangle represents what is shown in the main figures.

Figure S1

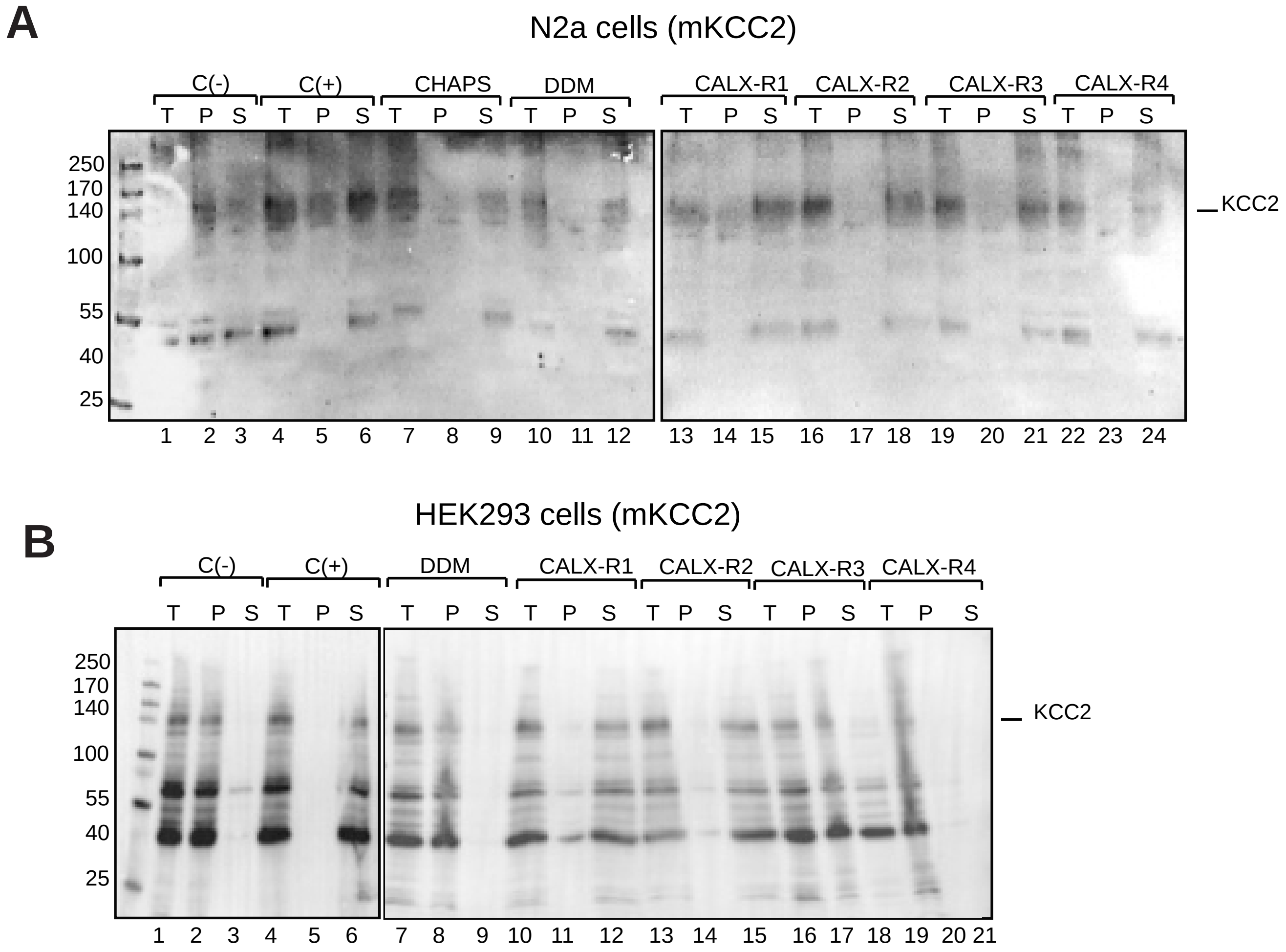
A
N2a cells (mKCC2)
C(-)
C(+)
CHAPS
DDM
CALX-R1
CALX-R2
CALX-R3
CALX-R4
T P S T P S T P S T P S T P S T P S T P S T P S
250
170
140
100
55
40
25
KCC2
1 2 3 4 5 6 7 8 9 10 11 12 13 14 15 16 17 18 19 20 21 22 23 24
B
HEK293 cells (mKCC2)
C(-)
C(+)
DDM
CALX-R1
CALX-R2
CALX-R3
CALX-R4
T P S T P S T P S T P S T P S T P S T P S
250
170
140
100
55
40
25
KCC2
1 2 3 4 5 6 7 8 9 10 11 12 13 14 15 16 17 18 19 20 21

Figure S2

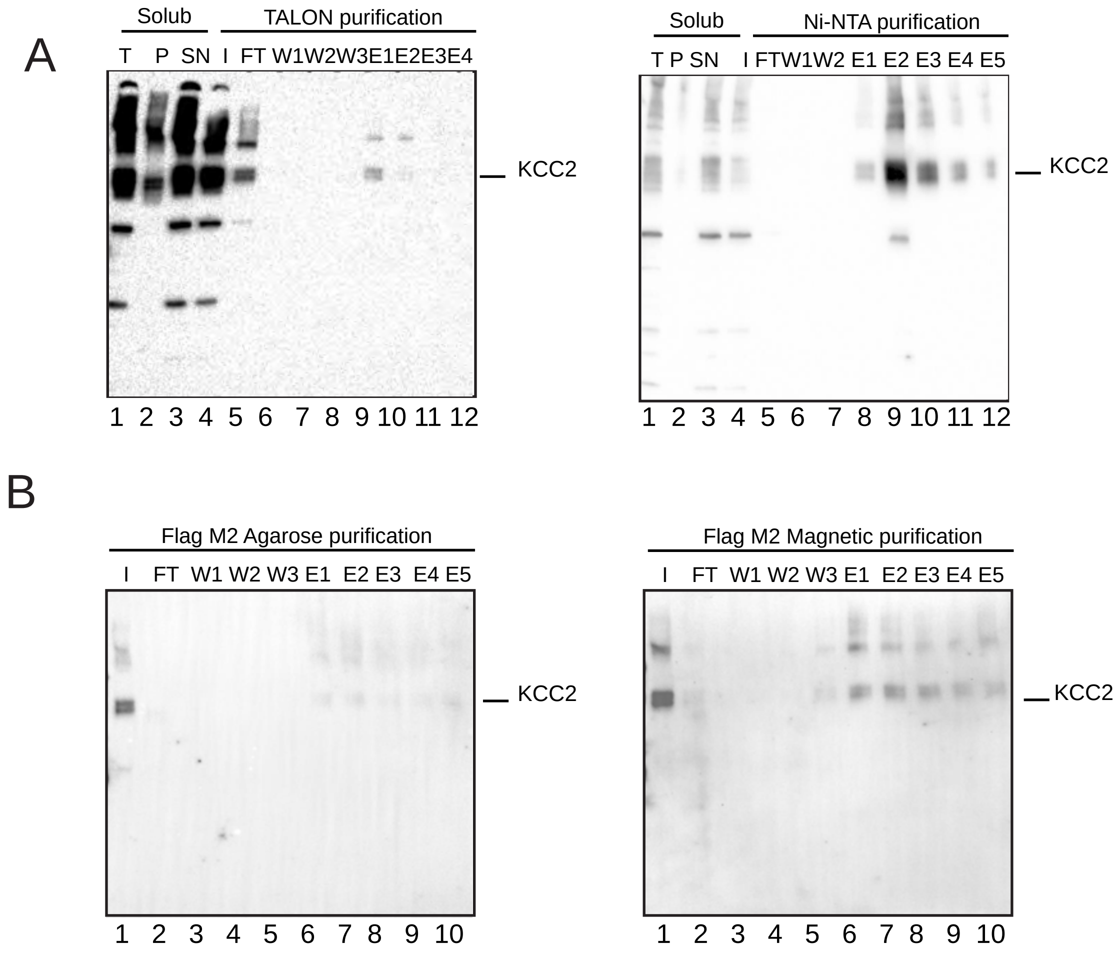
A
Solub
TALON purification
T P SN I FT W1W2W3E1E2E3E4
KCC2
1 2 3 4 5 6 7 8 9 10 11 12
Solub
Ni-NTA purification
T P SN I FTW1W2 E1 E2 E3 E4 E5
KCC2
1 2 3 4 5 6 7 8 9 10 11 12
B
Flag M2 Agarose purification
I FT W1 W2 W3 E1 E2 E3 E4 E5
KCC2
1 2 3 4 5 6 7 8 9 10
Flag M2 Magnetic purification
I FT W1 W2 W3 E1 E2 E3 E4 E5
KCC2
1 2 3 4 5 6 7 8 9 10

Figure S3

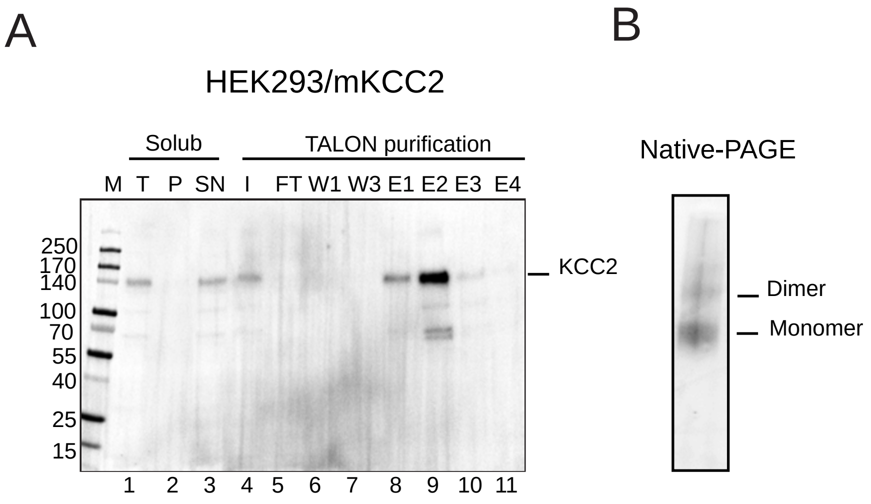
A
HEK293/mKCC2
Solub
TALON purification
M T P SN I FT W1 W3 E1 E2 E3 E4
250
170
140
100
70
55
40
25
15
KCC2
1 2 3 4 5 6 7 8 9 10 11
B
Native-PAGE
Dimer
Monomer

<u>Table S4:</u> List of experimental post-translational modifications (PTMs) found on KCC2 purified sample in comparison to previously reported work

PTMs	Amino-Acid (KCC2b)	Peptide MS detection	Literature		
			Species; Homologues	Technic	Source
Glycosylation	N283	AFDPPnFPICLLGNR			
	N291	SAFDPPNFPICLLGnR	Mouse KCC4	Mutation (Align with N312 of KCC4)	Weng et al. BBA (2013)
	N310	LAWEGnETVTTR	Mouse KCC4	Mutation (Align with N331 of KCC4)	Weng et al. BBA (2013)
	N328	FLnATCDEYFT			
	N338	nNVTEIQGIPGAASGLIK			
	N339	NnVTEIQGIPGAASGLIK	Mouse KCC4	Mutation (Align with N360 of KCC4)	Weng et al. BBA (2013)
Phosphorylation (Phosphosite, Hornbeck et al. Nucleid Acid Research (2012))	S25	EsSPFINSTDTEK	Mouse KCC2	MS	Wiśniewski JR, et al. *J Proteome Res* (2010).
	S26	ESsPFINSTDTEK	Mouse KCC2	MS	Wiśniewski JR, et al. *J Proteome Res* (2010).
	S940	EIQSITDESRGssIR	Human KCC2	WB	Kahle et al. EMBO Rep. (2014)
	T1007	VHLtWTK	Human KCC2	MS	Rinehart et al. Cell (2009)
	S1022	NKGPsPVSSEGIK	Rat KCC2	MS	Lundby A, et al. Nat. Commun.(2012)

Figure S5 : Sequence coverage of peptides (44 % of full length) of S12A5_HUMAN protein identified by LC-MSMS

Peptides identified with FDR <1% are highlighted in green, Peptides identified with FDR between 1 to 5% are highlighted in yellow.

MLNNLTDCED GDGGANPGDG NPK**ESSPFIN STDTEKGK**EY DGKNMALFEE EMDTSPMVSS LLSGLANYTN LPQGSR**EHEE**
AENNEGGKKK PVQAPRMGTF MGVYLPCLQN IFGVILFLRL TWVVGIAGIM ESFCMVFICC SCTMLTAISM SAIATNGVVP
AGGSYYMISR SLGPEFGGAV GLCFYLGTTF AGAMYILGTI EILLAYLFPA MAIFK**AEDAS GEAAAMLNNM R**VYGTCVLTC
MATVVFVGVK YVNKFALVFL GCVILSILAI YAGVIK**SAFD PPNFPICLLG NR**TLSR**HGFD VCAKLAWEGN ETVTTR**LWGL
FCSSR**FLNAT CDEYFTRNNV TEIQGIPGAA SGLIKENLWS SYLTK**GVIVE RSGMTSVGLA DGTPIDMDHP YVFSDMTSYF
TLLVGIYFPS VTGIMAGSNR **SGDLRDAQK**S IPTGTILAIA TTSAVYISSV VLFGACIEGV VLRDKFGEAV NGNLVVGTLA
WPSPWVIVIG SFFSTCGAGL QSLTGAPR**LL QAISRDGIVP FLQVFGHGK**A NGEPTWALLL TACICEIGIL IASLDEVAPI
LSMFFLMCYM FVNLACAVQT LLRTPNWRPR FRYYHWTLSF LGMSLCLALM FICSWYYALV AMLIAGLIYK YIEYR**GAEKE**
WGDGIRGLSL SAARYALLRL EEGPPHTKNW RPQLLVLVRV DQDQNVVHPQ LLSLTSQLKA GKGLTIVGSV LEGTFLENHP
QAQRAEESIR R**LMEAEKVKG FCQVVISSNL RDGVSHLIQS GGLGGLQHNT VLVGWPR**NWR **QKEDHQTWRN FIELVRETTA**
GHLALLVTKN VSMFPGNPER FSEGSIDVWW IVHDGGMLML LPFLLRHHKV WRKCKMR**IFT VAQMDDNSIQ MKKDLTTFLY**
HLRITAEVEV VEMHESDISA YTYEKTLVME QRSQILKQMH LTKNER**EREI QSITDESRGS IRRKNPANTR** LRLNVPEETA
GDSEEKPEEE VQLIHDQSAP SCPSSSPSPG EEPEGEGETD PEKVHLTWTK **DKSVAEKNKG PSPVSSEGIK DFFSMKPEWE**
NLNQSNVRRM HTAVR**LNEVI VKK**SRDAK**LV LLNMPGPPRN RNGDENYMEF LEVLTEHLDR VMLVR**GGGRE VITIYS

Figure S6 : PTM Mass spectrometry data of KCC2

Phosphorylation S25 EsSPFINSTDTEK

phosphoRS Site Probabilities : S(2): 50.0; S(3): 50.0; S(8): 0.0; T(9): 0.0; T(11): 0.0

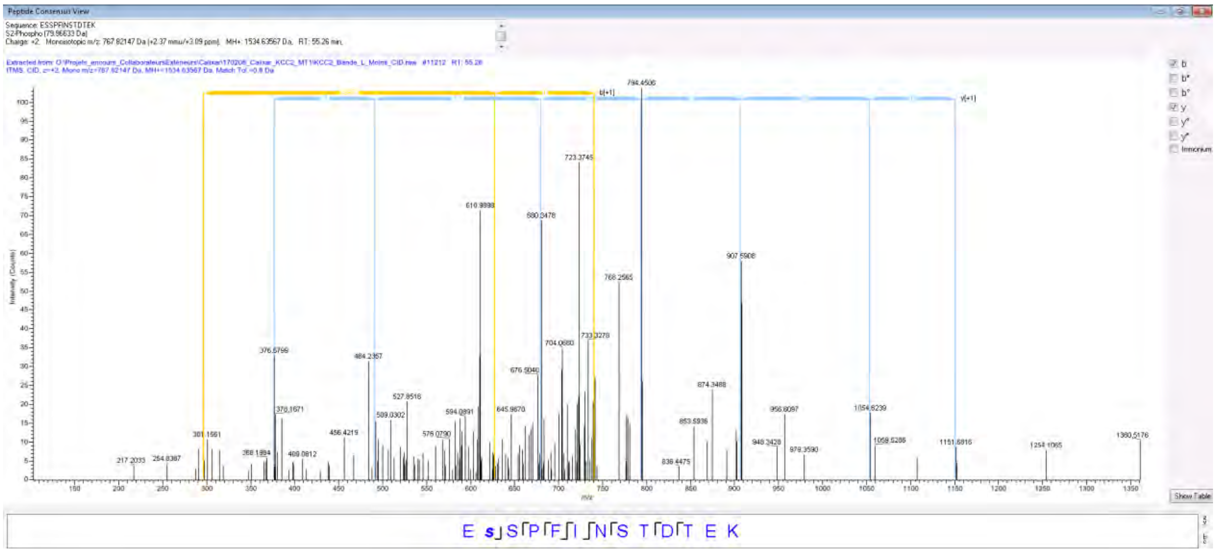

Phosphorylation S26 ESsPFINSTDTEK

phosphoRS Site Probabilities

S(2): 1.2; S(3): 98.8; S(8): 0.0; T(9): 0.0; T(11): 0.0

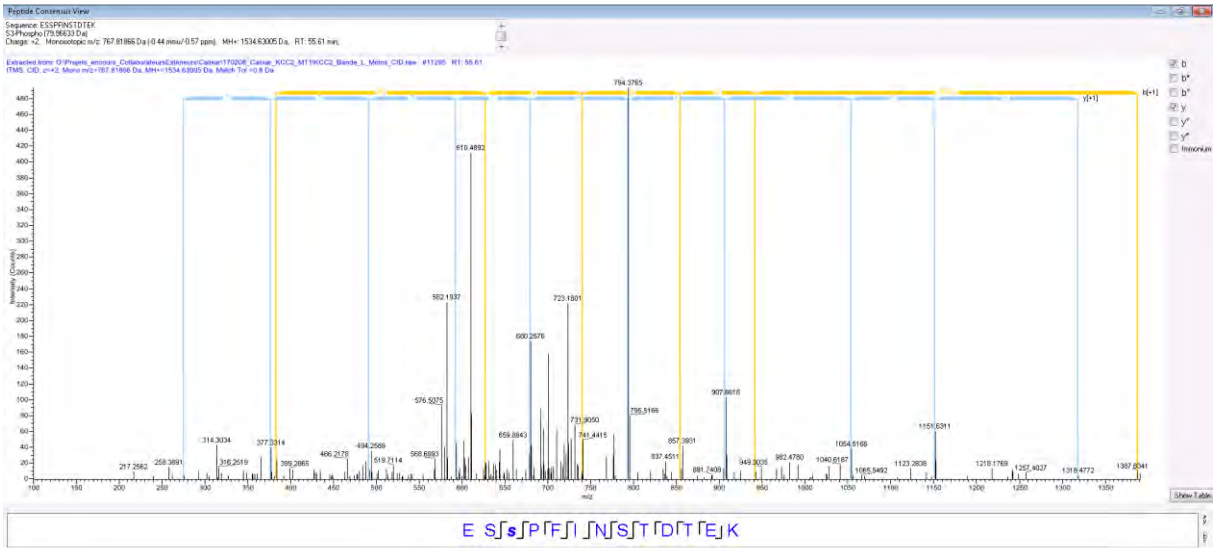

Phosphorylation T1007 VHLtWTK

phosphoRS Site Probabilities : T(4): 91.8; T(6): 8.2

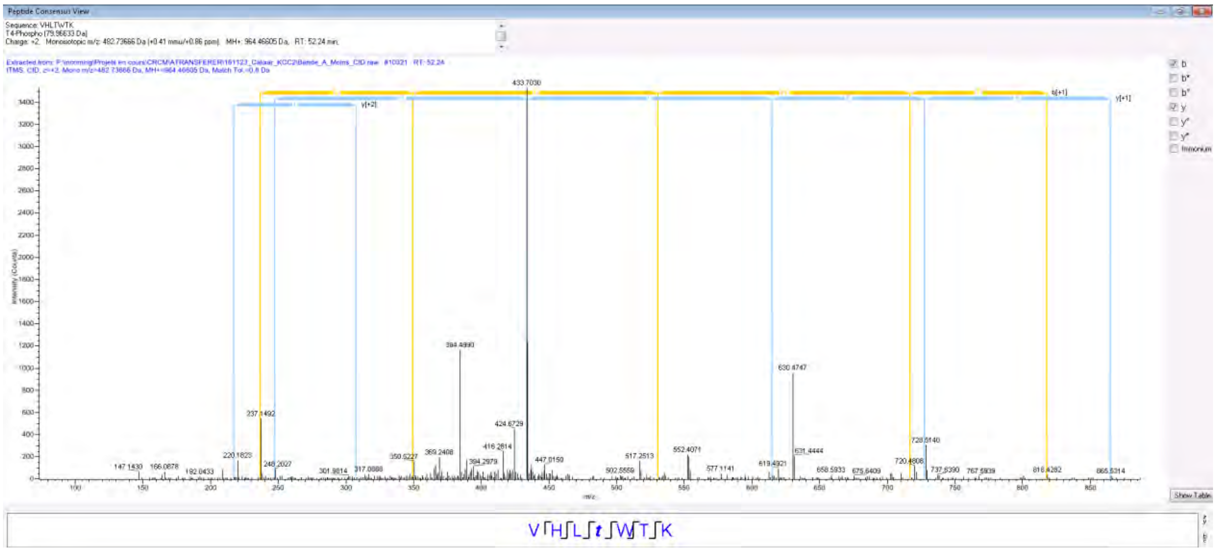

Phosphorylation S1022 NKGPsPVSSEGIK

phosphoRS Site Probabilities

S(5): 79.9; S(8): 10.1; S(9): 10.1

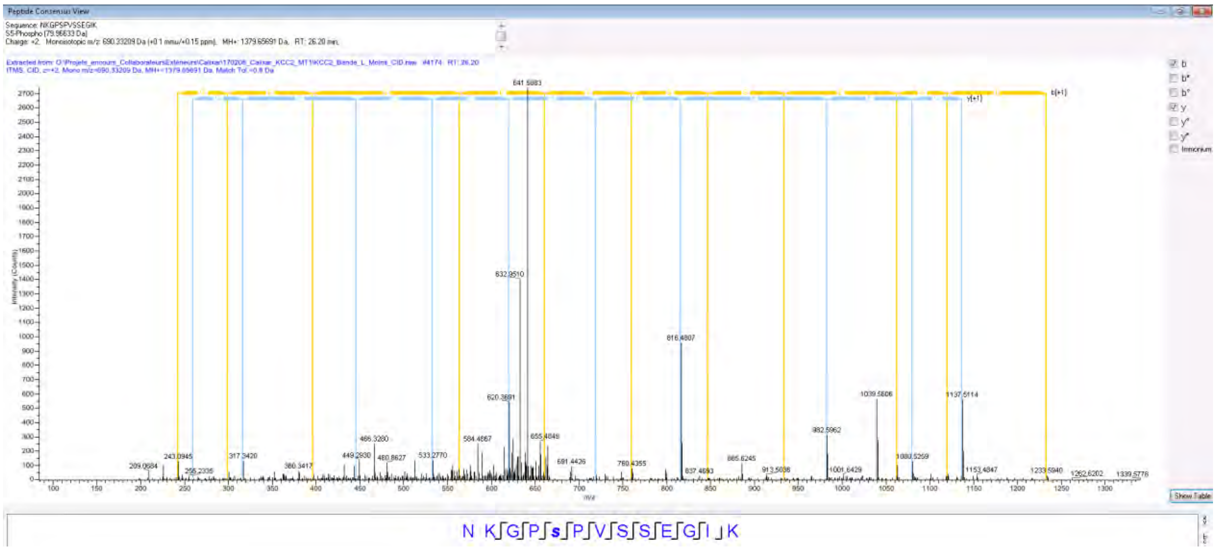

Phosphorylation S1022 GPsPVSSEGIK

phosphoRS Site Probabilities

S(3): 100.0; S(6): 0.0; S(7): 0.0

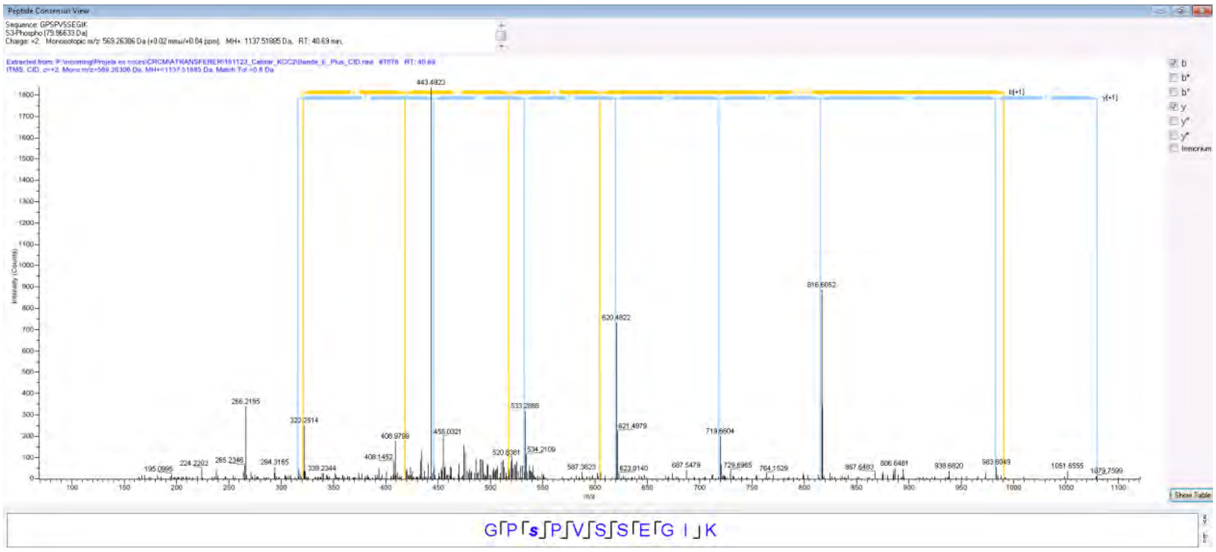

Phosphorylation S940 EIQSITDESRGsIR

phosphoRS Site Probabilities

S(4): 0.0; T(6): 0.0; S(9): 0.1; S(12): 99.9

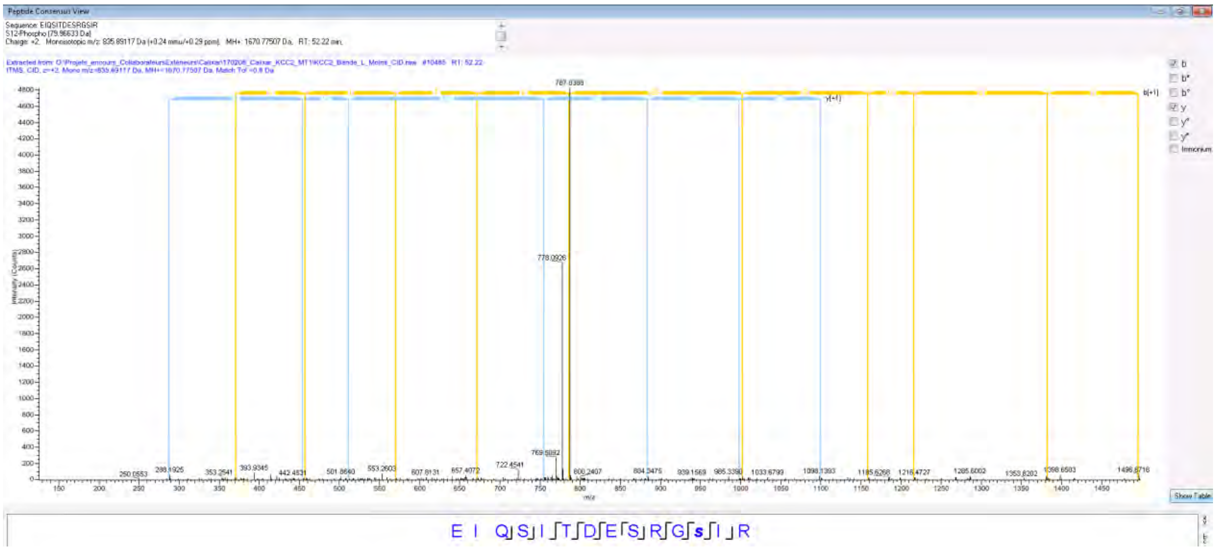

Glycosylation

N283 SAFDPPnFPICLLGNR

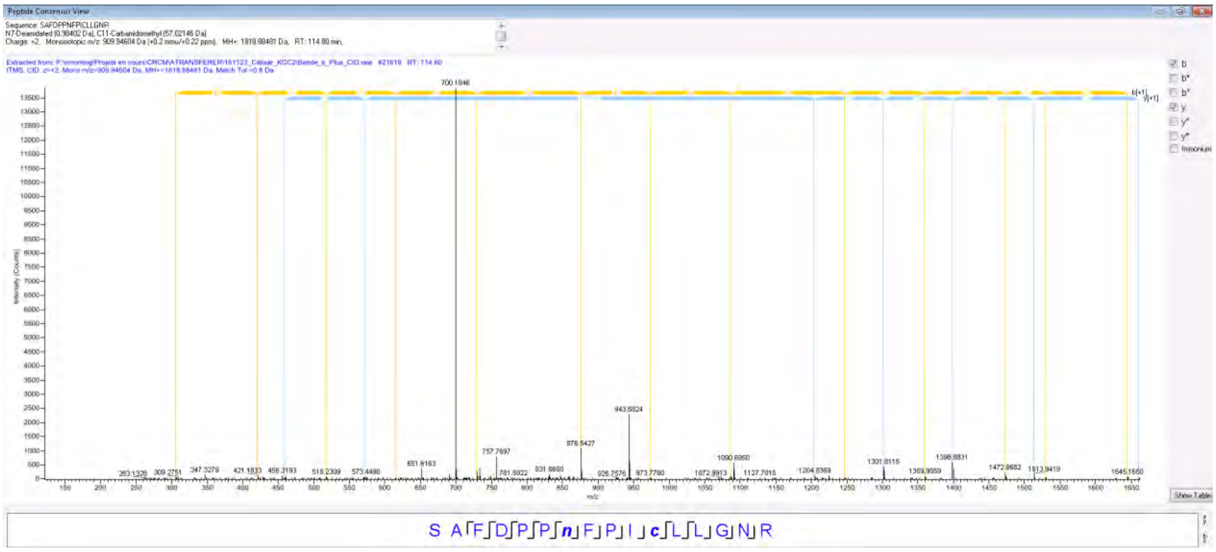

Glycosylation

N291 SAFDPPNFPICLLGnR

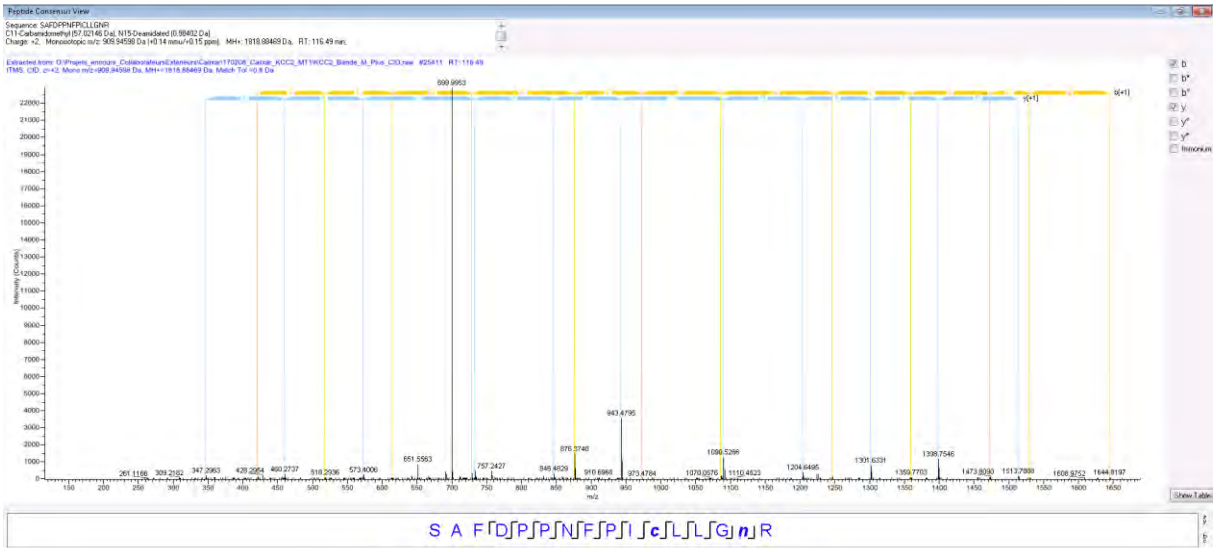

Glycosylation

N310 LAWEGnETVTTR

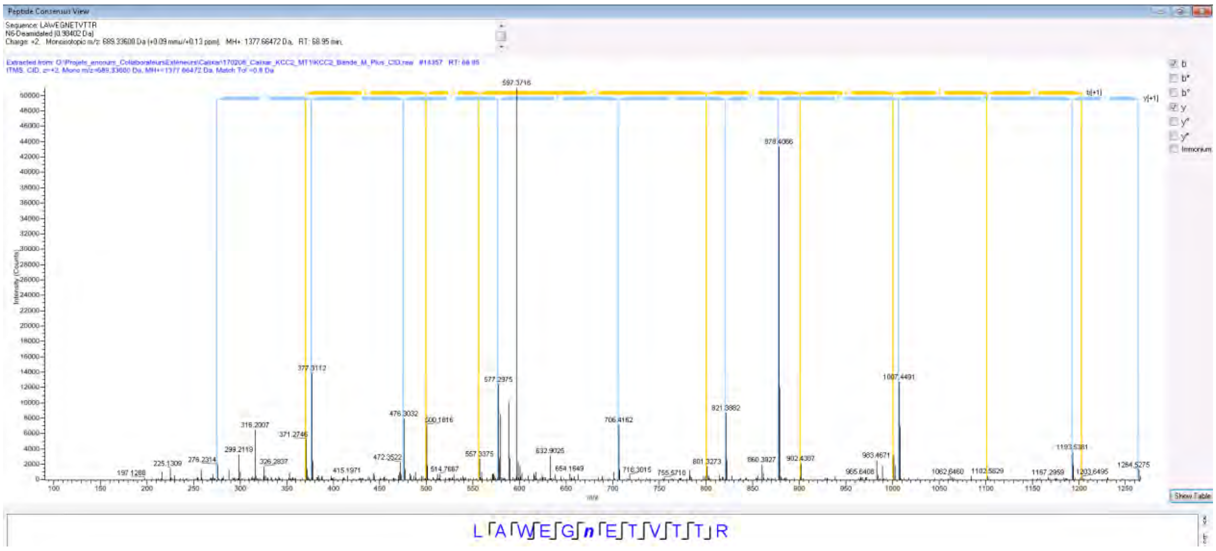

Glycosylation

N328 FLnATCDEYFTR

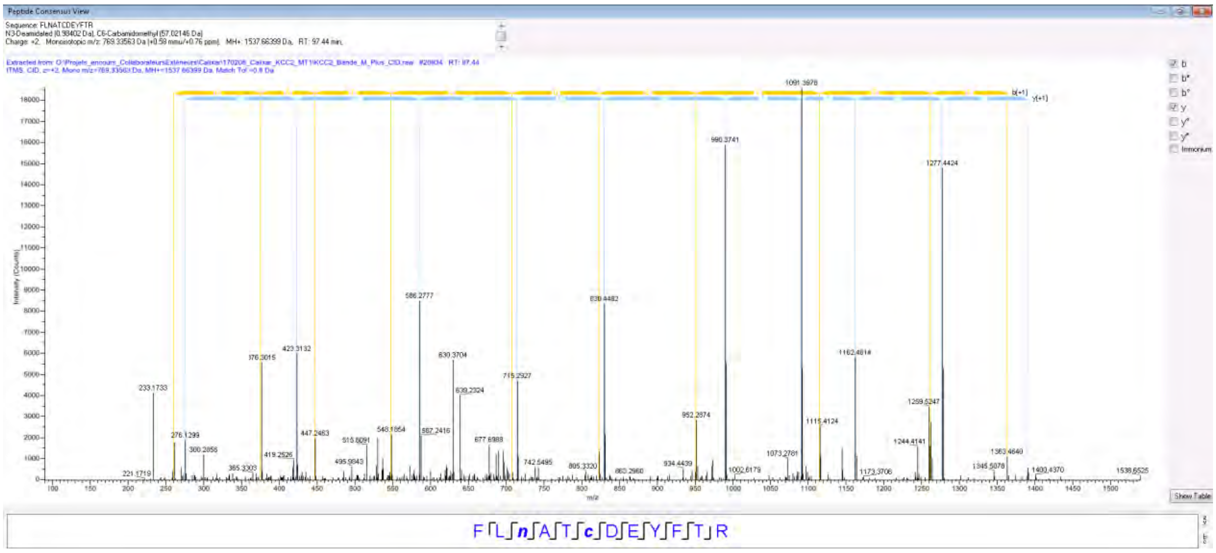

Glycosylation

N338 or N339 nnVTEIQGIPGAASGLIK

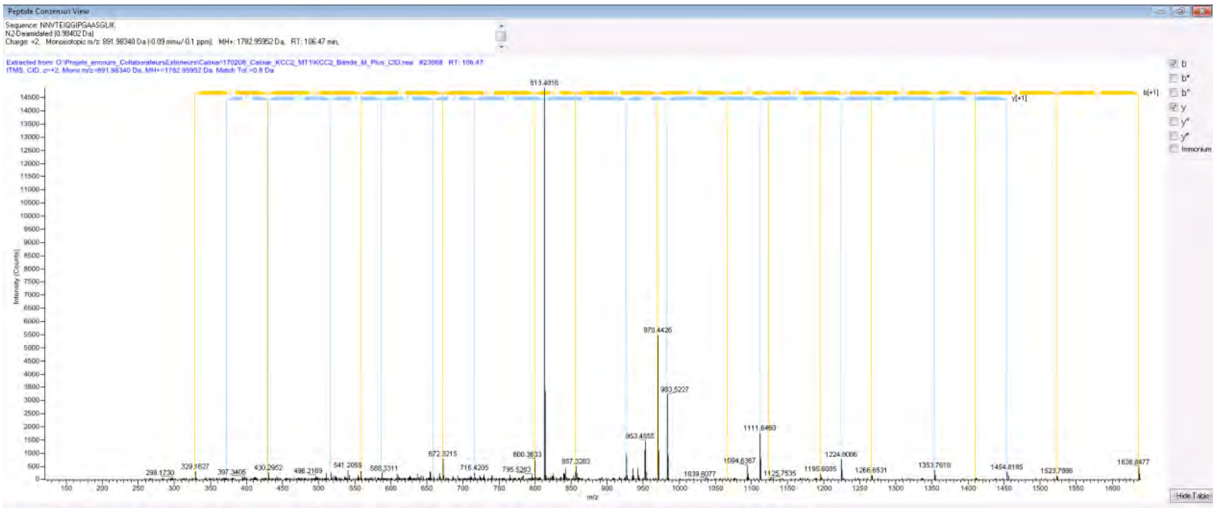

	(NH3+)-	n>	<V V>	<T T>	<E E>	<I I>	<Q Q>	G>	<I I>	<P P>	<G G>	<A A>	<A A>	<S S>	<G G>	<L L>	<I I> <K	K-(COOH)
b (+1)	115.05321	230.07715	329.14557	430.19325	559.23585	672.31992	800.37850	857.39997	970.48404	1067.53681	1124.55828	1195.59540	1266.63252	1353.66455	1410.68602	1523.77009	1636.85416	1764.94913
b*(+1)	98.02366	213.05060	312.11902	413.16670	542.20930	655.29337	783.35195	840.37342	953.45749	1050.51026	1107.53173	1178.56885	1249.60597	1336.63800	1393.65947	1506.74354	1619.82761	1747.92258
b°(+1)	97.03964	212.06659	311.13501	412.18269	541.22529	654.30936	782.36794	839.38941	952.47348	1049.52625	1106.54772	1177.58484	1248.62196	1335.65399	1392.67546	1505.75953	1618.84360	1746.93857
y (+1)	1668.91677	1553.88982	1454.82140	1353.77372	1224.73112	1111.64705	983.58847	926.56700	813.48293	716.43016	659.40869	588.37157	517.33445	430.30242	373.28095	260.19688	147.11281	1782.95970
y*(+1)	1651.89022	1536.86327	1437.79485	1336.74717	1207.70457	1094.62050	966.56192	909.54045	796.45638	699.40361	642.38214	571.34502	500.30790	413.27587	356.25440	243.17033	130.08626	1765.93315
y°(+1)	1650.90620	1535.87926	1436.81084	1335.76316	1206.72056	1093.63649	965.57791	908.55644	795.47237	698.41960	641.39813	570.36101	499.32389	412.29186	355.27039	242.18632	129.10225	1764.94913

Figure S7

A

Monomer

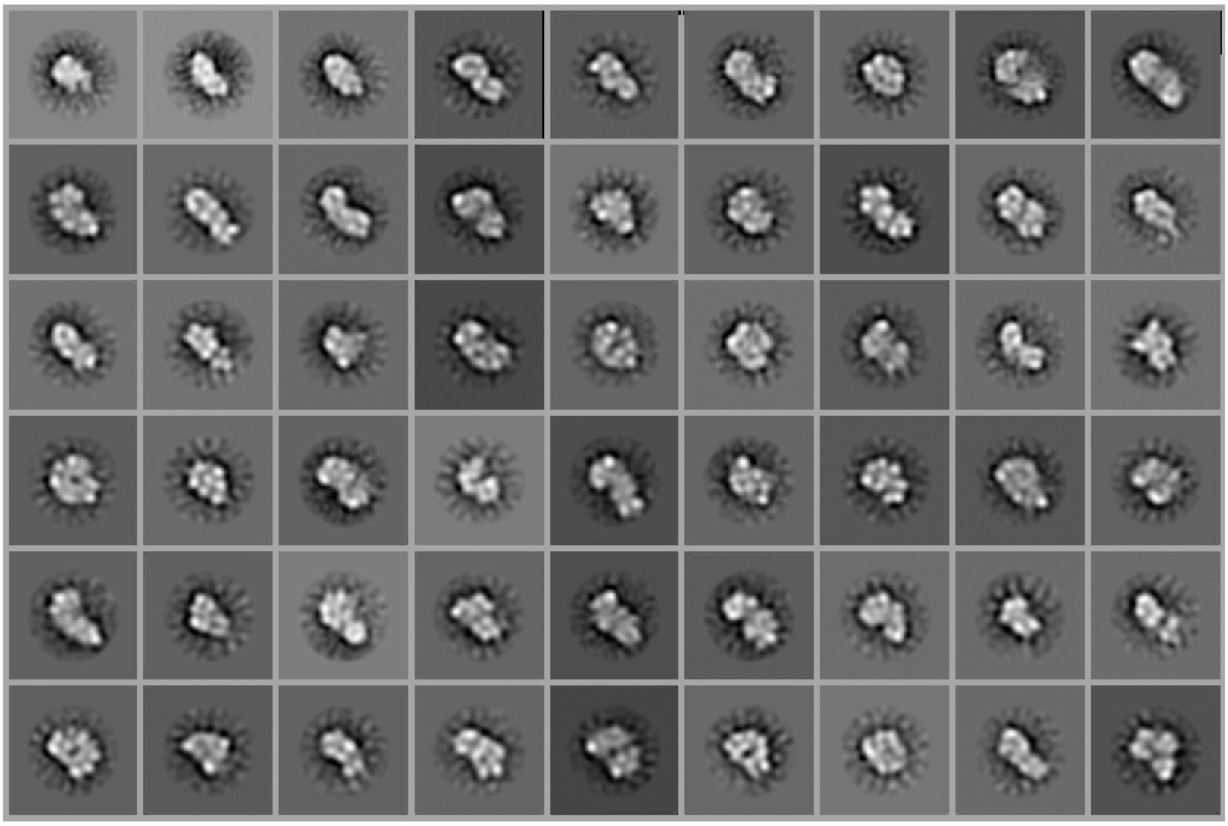

B

Dimer

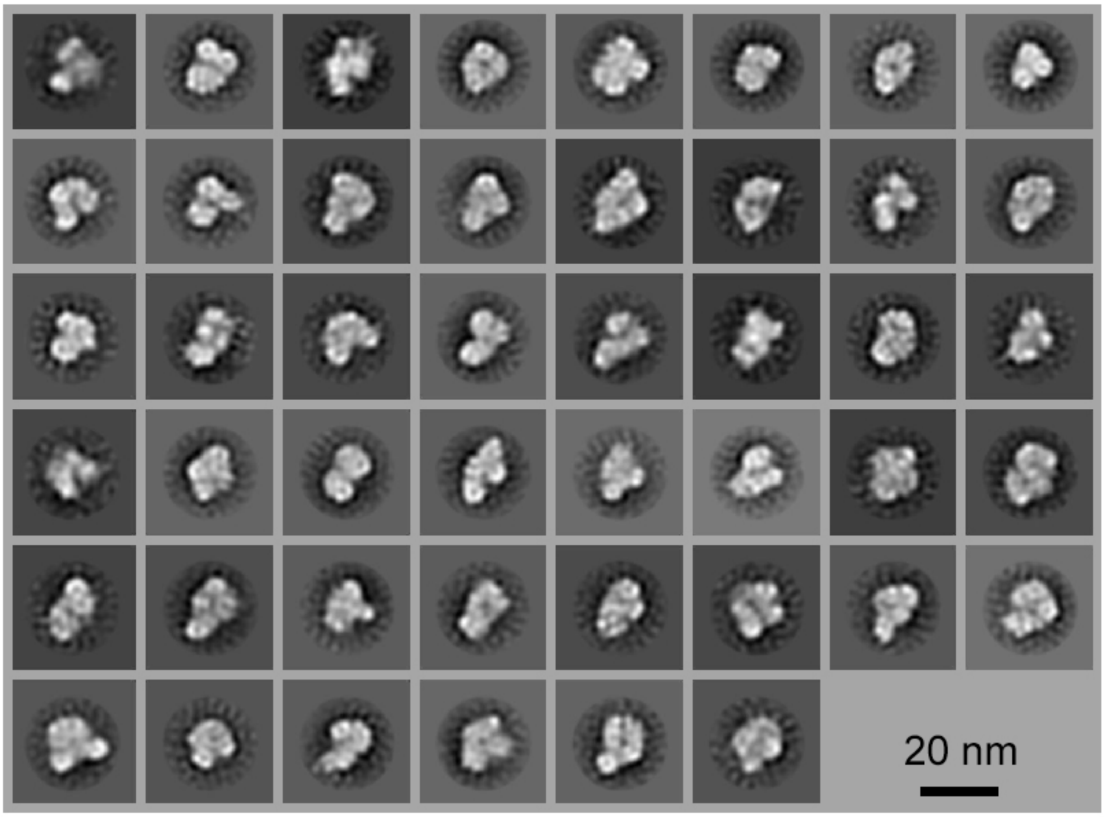

Figure S10

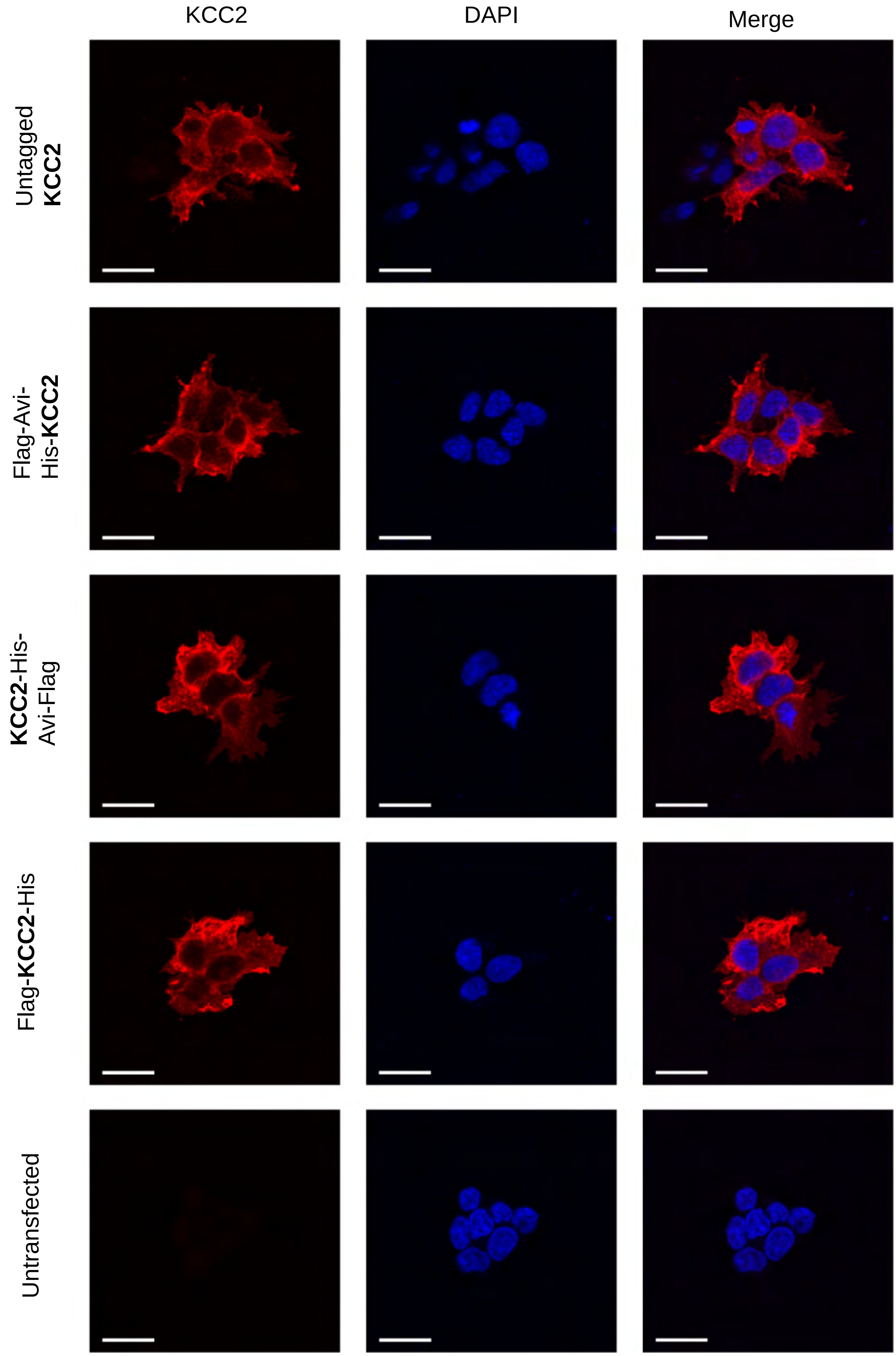

KCC2
DAPI
Merge
Untagged
KCC2
Flag-Avi-
His-KCC2
KCC2-His-
Avi-Flag
Flag-KCC2-His
Untransfected

Figure S11

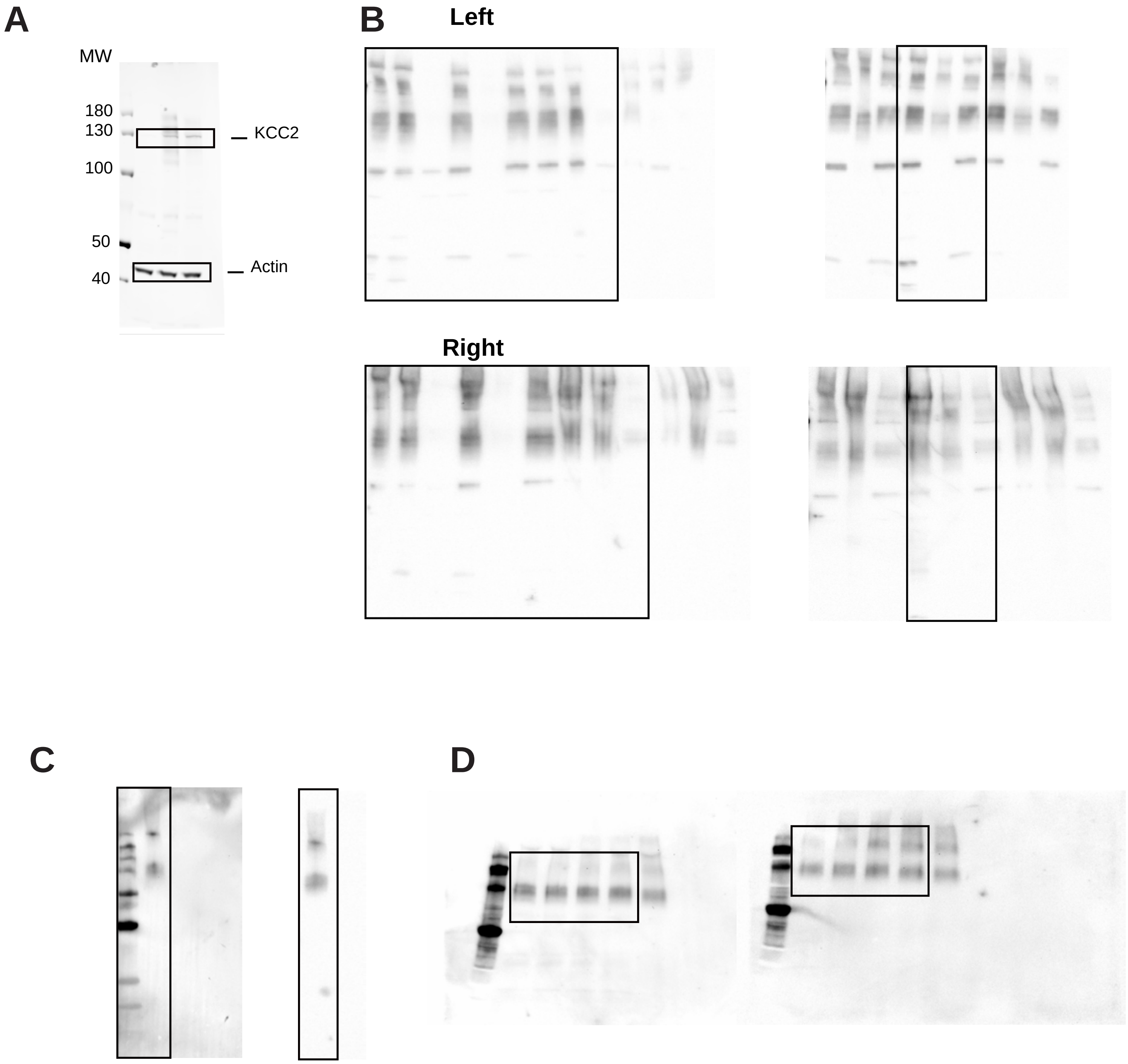

Supplement: Supplementary file 1 — Supplementary Information [file 41598_2017_15739_MOESM1_ESM.pdf]
